# Mitigating digestive disorders: Action mechanisms of Mediterranean herbal active compounds

**DOI:** 10.1515/biol-2022-0857

**Published:** 2024-04-18

**Authors:** Abdalsalam Kmail

**Affiliations:** Faculty of Sciences, Arab American University Jenin, P. O. Box 240, Jenin, Palestine

**Keywords:** Mediterranean diet, active compounds, digestive disorders

## Abstract

This study explores the effects of the Mediterranean diet, herbal remedies, and their phytochemicals on various gastrointestinal conditions and reviews the global use of medicinal plants for common digestive problems. The review highlights key plants and their mechanisms of action and summarizes the latest findings on how plant-based products influence the digestive system and how they work. We searched various sources of literature and databases, including Google Scholar, PubMed, Science Direct, and MedlinePlus. Our focus was on gathering relevant papers published between 2013 and August 2023. Certain plants exhibit potential in preventing or treating digestive diseases and cancers. Notable examples include *Curcuma longa, Zingiber officinale, Aloe vera, Calendula officinalis, Lavandula angustifolia, Thymus vulgaris, Rosmarinus officinalis, Ginkgo biloba, Cynodon dactylon*, and *Vaccinium myrtillus*. The phytochemical analysis of the plants showed that compounds such as quercetin, anthocyanins, curcumin, phenolics, isoflavones glycosides, flavonoids, and saponins constitute the main active substances within these plants. These natural remedies have the potential to enhance the digestive system and alleviate pain and discomfort in patients. However, further research is imperative to comprehensively evaluate the benefits and safety of herbal medicines to use their active ingredients for the development of natural and effective drugs.

## 1 Introduction

**Figure 1:**
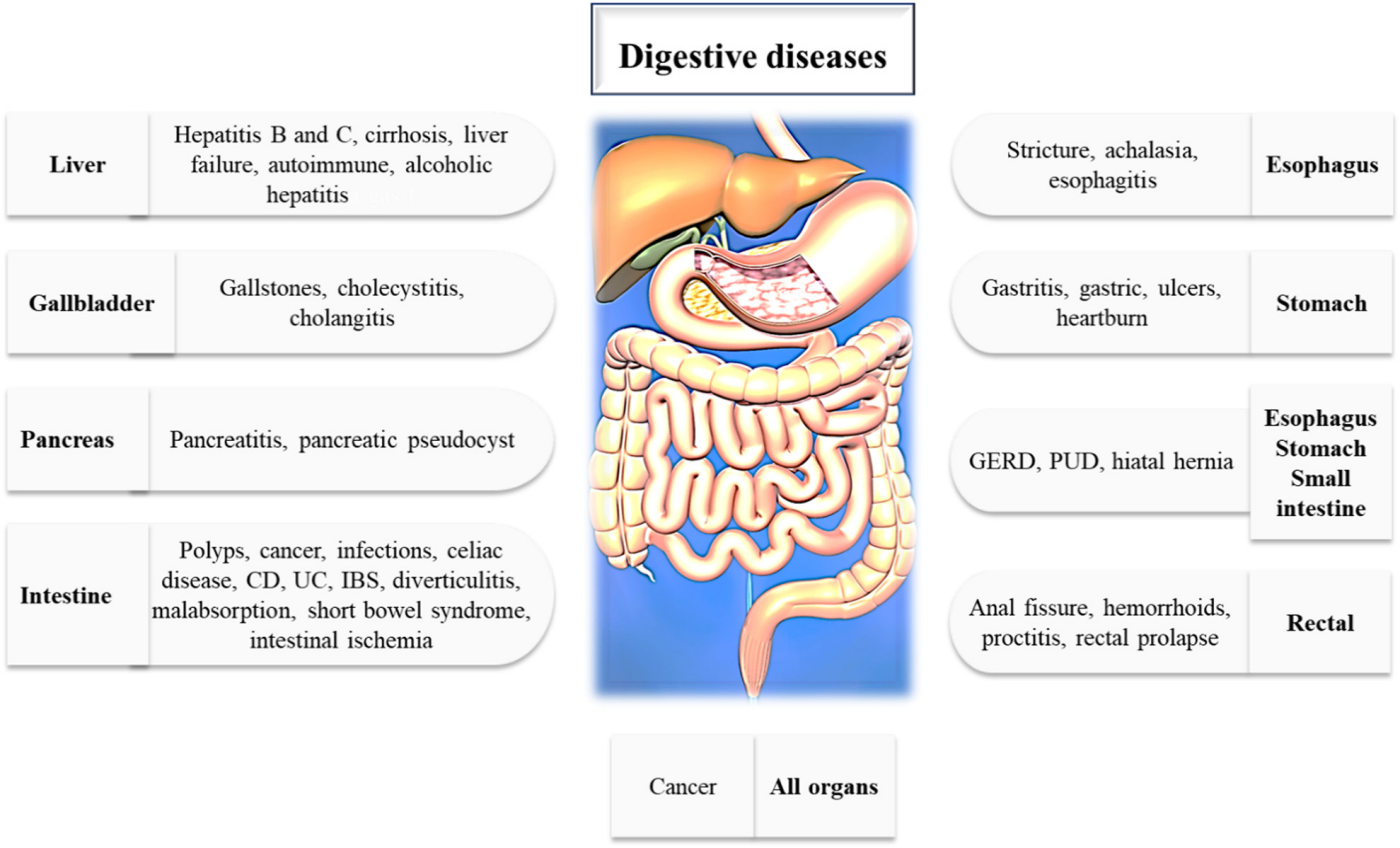
Common digestive disorders. CD: Crohn’s disease; UC: ulcerative colitis; IBS: irritable bowel syndrome; GERD: gastroesophageal reflux disease; PUD: peptic ulcer disease.

Globally, digestive disorders are widespread, often frustrating, and occasionally life threatening. In 2019, digestive disorders accounted for 2276.27 million predicted prevalent cases, 2.56 million fatalities, and 88.99 million disability-adjusted life-years worldwide [1]. Some digestive disorders and ailments are acute, lasting only a short period, while others are chronic, persisting over the long term. Common symptoms of digestive disorders, including bleeding, bloating, constipation, diarrhea, heartburn, incontinence, abdominal discomfort, swallowing, weight gain or loss, nausea, and vomiting [2]. Regrettably, most people suffer from gut and digestive system issues virtually from birth. Common digestive disorders include gastroesophageal reflux disease (GERD), irritable bowel syndrome (IBS), inflammatory bowel disease (IBD), colorectal cancer (CRC), peptic ulcer disease (PUD), as well as lactose intolerance, hiatal hernia, liver disease (LD), pancreatitis, heartburn, and cancer (Figure 1) [3]. While conventional medications are commonly used to treat digestive disorders, they come with inherent risks. These drugs often yield adverse effects such as nausea, vomiting, and diarrhea. Moreover, certain oral medications may negatively impact the digestive tract, leading to esophageal strictures, ulcers, bleeding, and constriction. When multiple medications are administered concurrently, the potential for interactions increases significantly. These interactions can lead to adverse effects, including food allergies, sensitivities, and exacerbation of existing conditions such as diabetes, renal illness, or LD [4,5]. Alternative medicine offers a wealth of treatment possibilities. Natural substances and their structural analogs have significantly influenced pharmacotherapy in the past. The quest for scaffolds exhibiting a wide range of bioactivities and substantial structural diversity remains feasible through the use of natural products. These scaffolds can be either directly produced or serve as building blocks for the development of novel drugs. However, natural products face persistent challenges due to high attrition rates in medication research. Factors such as sustainable supply, accessibility, and intellectual property restrictions add complexity to their utilization. Fortunately, recent scientific and technological advancements are addressing these concerns and opening up new opportunities [6].

The Mediterranean flora includes numerous aromatic and diverse medicinal plants that have long been an integral part of local culture, representing a wide range of ecological contexts [7,8]. Among the plants that are traditionally used in the Mediterranean Basin, species from the Lamiaceae, Asteraceae, and Apiaceae families are the most prevalent. Notably, some of these plants have already been examined for their pharmacological properties, including sage, rosemary, thyme, oregano, lavender, and other Lamiaceae species [9]. Natural bioactive compounds have been used for food and medicine purposes since prehistoric times. People have employed plants in traditional recipes to improve nutrition and overall health. Interestingly, many contemporary medications are rooted in the traditional knowledge of plant-based medicinal potential. The overwhelming majority of the population (approximately 80%, particularly in developing countries) continues to rely on plant-based traditional medicines for basic health care needs [10]. These digestive plant preparations operate through various mechanisms, including healing the intestine lining, activating the mechanical and chemical digestion, as well as promoting smooth bowel movements, enhancing bowel frequency, detoxifying and eliminating toxins, and soothing upset stomach. Furthermore, they play a role in reducing gas, bloating, and other digestive problems. Medicinal plants likely represent the earliest form of medication, having been utilized by diverse societies throughout history. They have been utilized by many societies throughout history and continue to be an important element of our current technological advanced society. Plant-derived pharmaceuticals or natural compounds and their derivatives account for over than half of all clinically used medications [11].

Medicinal plants and phytomedicines offer several benefits, including their natural origin, minimal side effects, and avoidance of dissatisfaction associated with conventional medication [12]. A recent study provides compelling evidence that various functional food types such as probiotics, prebiotics, and herbs may have a favorable impact on the digestive tract [13]. Probiotics are likely to be beneficial in the prevention of IBD, whereas prebiotics include fibers that may inhibit the development of IBD [14,15,16]. Triangles, elm, and pie plant are herbs that may be associated with alleviating symptoms of IBDs. Mastic, herbal origin, may help manage heartburn, which is a common symptom of GERD [17]. Blueberries have been shown to lessen diarrhea, a frequent sign of gastrointestinal illnesses [18]. A double-blind experiment revealed that *Aloe vera* alleviated symptoms of persistent constipation [19]. In addition, *A. vera* syrup showed promise in managing GERD in a preliminary randomized positive-controlled experiment [20]. The bioactive components of Mediterranean diet (MD) known for their health benefits found to reduce chronic inflammation by influencing C-reactive protein, interleukin-6 (IL-6), and tumor necrosis factor-alpha (TNF-α) [21]. Recent analysis suggests that inflammation-induced platelet-activating factor plays a pivotal role in initiating cardiovascular diseases (CVD) rather than a mere rise in blood cholesterol levels [22]. Given the link between diet and gastrointestinal health, further research is needed to explore the potential impact of specific functional foods and bioactive substances synergistically present in the MD [23]. More clinical and epidemiological studies are essential to unravel the intricate role of nutrition and bioactive chemicals in gastrointestinal illnesses to promote the consumption of functional foods [23].

The purpose of the current review is to explore the diverse mechanisms of action associated with herbal-based medicine and the MD, along with their active components, specifically concerning the digestive system. In addition, we highlight their potential advantages for human health, particularly in the prevention and treatment of digestive disorders such as GERD, IBS, IBD, CRC, and PUD. Our focus lies on identifying the most prevalent and effective active compounds and compiling a list of important medicinal herbs.

## 2 MD

The traditional MD emerged in the nations of the Mediterranean Basin, where a warm and pleasant environment supports the cultivation of a wide variety of fruits and vegetables all year. Consequently, the MD is characterized by an abundance of these fruits and vegetables, along with the prominent use of extra virgin olive oil as the primary source of fat. Legumes, whole grains, nuts, seeds, and fragrant herbs also play a vital role in this dietary pattern [24,25]. Today, the phrase “MD” broadly refers to the typical eating practices of nations bordering the Mediterranean Sea. However, it is essential to recognize that historically, the Greek term “diaita” referred to more than just daily dietary habits. It encompassed a complete way of life, aligning with modern concepts of lifestyle and well-being [26]. Recent studies demonstrate that these components may have health advantages such as protection against and prevention of significant health issues such as diabetes, CVD, skin disease, digestive disease, and aging [10,27]. Active substances derived from MD such as polyphenols, omega-3 fatty acids, carotenoids, vitamins, folate, magnesium, aromatic compounds (e.g., rosmarinic acid), and curcumin are proven for their protective as well as therapeutic effects on the aforementioned disorders (Table 1). Interestingly, plants synthesize an estimated 100,000 of these organic compounds, yet only 10% of them have been identified. These metabolites fall into three categories: terpenes, phenolics, and alkaloids (nitrogen-containing chemicals), each with therapeutic effects. Flavonoids, found in blueberries, strawberries, grapes, melons, citrus fruits, apricots, onions, cabbage, fennel, tomatoes, lettuce, broccoli, spinach, and other foods, as well as frequent yogurt eating appear to reduce the probability of developing several tumors [24,25]. The active compounds found in medicinal and aromatic plants vary greatly based on the portion of the plant (inflorescence, bracts, leaves, stems), the developmental stage, the season, the time of day, and the environmental factors that influenced the cultivation or natural growth of an aromatic-medicinal plant [28,29].

**Table 1:** Active compounds from MD with their target mechanisms in various diseases

Active compound	Plant source	Disease	Target	References
Polyphenols	Olive oil, fruits, vegetables, nuts, legumes, cereals	Obesity, diabetes, CVD, cancer, neurodegenerative disease, digestive diseases, skin diseases	Antioxidant, anti-inflammatory, anti-diabetic, anti-atherogenic, anti-cancer, neuroprotective, gastroprotective, dermatoprotective	[30,31]
Omega-3 fatty acids	Fish, nuts, seeds	Obesity, diabetes, CVD, cancer, neurodegenerative disease, digestive diseases, skin diseases	Anti-inflammatory, anti-diabetic, anti-atherogenic, anti-cancer, neuroprotective, gastroprotective, dermatoprotective	[30]
Fiber	Fruits, vegetables, nuts, legumes, cereals	Obesity, diabetes, CVD, cancer, digestive diseases	Prebiotic, satiating, anti-diabetic, anti-atherogenic, anti-cancer, gastroprotective	[32]
Carotenoids	Fruits, vegetables	Obesity, diabetes, CVD, cancer, skin diseases	Antioxidant, anti-inflammatory, anti-diabetic, anti-atherogenic, anti-cancer, dermatoprotective	[33]
Vitamin C	Fruits, vegetables	Obesity, diabetes, CVD, cancer, skin diseases	Antioxidant6, anti-inflammatory, anti-diabetic, anti-atherogenic, anti-cancer, dermatoprotective5	[32,33]
Vitamin E	Olive oil, nuts, seeds	Obesity, diabetes, CVD, cancer, skin diseases	Antioxidant, anti-inflammatory, anti-diabetic, anti-atherogenic, anti-cancer, dermatoprotective	[32,33]
Folate	Fruits, vegetables	CVD	Anti-atherogenic	[32,33]
Magnesium	Nuts, seeds	CVD	Anti-hypertensive	[33,34]
Aromatic compounds	Rosemary	Obesity, cancer, skin diseases	Anti-obesity, anti-cancer, antimicrobial	[35]
Curcumin	Turmeric	Obesity, diabetes, CVD, cancer, neurodegenerative disease	Antioxidant, anti-inflammatory, anti-diabetic, anti-atherogenic, anti-cancer, neuroprotective	[35]

It is challenging to trace the origins of Mediterranean cuisine, although it most likely evolved alongside cultures inhabiting the Mediterranean Basin since the birth of civilization. Over time, this culinary tradition absorbed influences from various invaders while preserving many local customs. The Fertile Crescent, the Near East geographical region, lies between the eastern tip of the Mediterranean Sea and the Persian Gulf, and it encompasses areas such as Mesopotamia, Canaan, and some argue, Northern Egypt. These regions may well had contributed to the rich tapestry of Mediterranean cuisine [36].

## 3 Digestive diseases

According to a recent survey published involving 73,000 adults across 33 countries on six continents, more than 40% of the global population experiences functional gastrointestinal disorders (FGIDs). Among these individuals, 49.5% of females and 36.6% of males meet eligibility for at least one of the FGIDs [37]. Digestive disorders influence approximately 40 million people in the United States leading to millions of clinical visits annually. The associated costs reached a staggering $119.6 billion in the year 2018 [38,39]. Furthermore, the burden of digestive disorders is elevating in developing countries [39]. These common digestive diseases that affect millions of people worldwide significantly affect the quality of life and contribute to morbidity and mortality. Accurate diagnosis and appropriate management, both pharmacological and nonpharmacological, are of utmost importance. Some of the main digestive diseases, their impact on work activities, and why they are important are addressed here.

### 3.1 GERD

GERD is a digestive disease characterized by the backflow of stomach acid or bile into the esophagus, leading to irritation and inflammation of its lining. The disrupting mechanism in the lower esophageal sphincter ordinarily prevents stomach contents from refluxing. Common symptoms of GERD include heartburn, chest discomfort, regurgitation, and trouble swallowing. GERD is a prevalent condition that affects people of all ages and genders, with global rates ranging from 8 to 33% [40]. GERD is mainly associated with proton pump inhibitor (PPIs) management [41]. PPI medications are often prescribed as an initial empirical diagnostic strategy for patients with typical GERD symptoms (such as heartburn and regurgitation) and atypical symptoms (such as noncardiac chest pain, chronic cough, hoarseness, throat clearing, and wheezing) [42,43]. Functional esophageal problems are mainly treated with neuromodulators, which alter the neuronal activity without acting as neurotransmitters. Various notable neuromodulators explored for managing functional esophageal issues, particularly noncardiac chest discomfort, such as tricyclic antidepressants, trazodone, selective serotonin reuptake inhibitors, and serotonin norepinephrine, reuptake inhibitors [44]. In addition, many Mediterranean plants, such as *Artemisia absinthium*, *Humulus lupulus*, *Matricaria recutita*, *Foeniculum vulgare*, and *Thymus vulgaris*, are well-known for their effects on GERD therapies. These plants contain active constituents that contribute to their therapeutic properties are listed in Table 2.

**Table 2:** Mediterranean plants and their active compounds used to treat specific digestive diseases, with their mechanisms of action

Plant	Active compound	Digestive disease	Mechanism of action	Reference
*Artemisia absinthium* (Wormwood)	Sesquiterpene lactones, flavonoids, phenolic acids	GERD, PUD	Antacid, anti-inflammatory, antibacterial, antispasmodic, cytoprotective	[45]
*Humulus lupulus* (Hops)	Alpha and beta acids, flavonoids, prenylated chalcones	GERD, PUD, IBS	Antacid, anti-inflammatory, antibacterial, antispasmodic, anxiolytic	[46]
*Ginkgo biloba* (Ginkgo)	Flavonoids, terpenoids	IBS, IBD, CRC	Anti-inflammatory, antioxidant, immunomodulatory, anticancer	[47]
*Panax* spp. (Ginseng)	Ginsenosides, polysaccharides, phenolic compounds	IBS, IBD, CRC, LD	Anti-inflammatory, antioxidant, immunomodulatory, anticancer, hepatoprotective	[48]
*Ganoderma lucidum* (Reishi mushroom)	Triterpenoids, polysaccharides, phenolic compounds	IBS, IBD, CRC, LD	Anti-inflammatory, antioxidant, immunomodulatory, anticancer, hepatoprotective	[49]
*Gynostemma pentaphyllum* (Jiaogulan)	Gypenosides, flavonoids, saponins	IBS, IBD, CRC, LD	Anti-inflammatory, antioxidant, immunomodulatory, anticancer, hepatoprotective	[46]
*Matricaria recutita* (Chamomile)	Flavonoids, terpenoids, coumarins, phenolic acids	GERD, PUD, IBS, IBD	Antacid, anti-inflammatory, antibacterial, antispasmodic, sedative	[50]
*Foeniculum vulgare* (Fennel)	Monoterpenes, phenylpropanoids, flavonoids, coumarins	GERD, PUD, IBS, IBD	Antacid, anti-inflammatory, antibacterial, antispasmodic, carminative	[51]
*Thymus vulgaris* (Thyme)	Phenolic monoterpenes, flavonoids, phenolic acids	GERD, PUD, IBS, IBD	Antacid, anti-inflammatory, antibacterial, antispasmodic, antioxidant	[52]
*Rosmarinus officinalis* (Rosemary)	Phenolic diterpenes, flavonoids, phenolic acids	GERD, PUD, IBS, IBD, CRC	Antacid, anti-inflammatory, antibacterial, antispasmodic, antioxidant, anticancer	[51]
*Salvia officinalis* (Sage)	Phenolic diterpenes, flavonoids, phenolic acids	GERD, PUD, IBS, IBD, CRC	Antacid, anti-inflammatory, antibacterial, antispasmodic, antioxidant, anticancer	[53]
*Origanum vulgare* (Oregano)	Phenolic monoterpenes, flavonoids, phenolic acids	GERD, PUD, IBS, IBD, CRC	Antacid, anti-inflammatory, antibacterial, antispasmodic, antioxidant, anticancer	[54]
*Cynodon dactylon* (Bermuda grass)	Flavonoids, phenolic acids, triterpenoids, steroids	PUD, IBD, CRC	Anti-ulcer, anti-inflammatory, antioxidant, anticancer	[55]
*Borago officinalis* (Borage)	Gamma-linolenic acid, rosmarinic acid, flavonoids	GERD, PUD, IBS, IBD	Antacid, anti-inflammatory, antibacterial, antispasmodic	[56]
*Lavandula angustifolia* (Lavender)	Linalool, linalyl acetate, terpenoids, flavonoids	GERD, PUD, IBS, IBD	Antacid, anti-inflammatory, antibacterial, antispasmodic, sedative	[50]
*Mentha piperita* (Peppermint)	Menthol, menthone, flavonoids, phenolic acids	GERD, PUD, IBS, IBD	Antacid, anti-inflammatory, antibacterial, antispasmodic, carminative	[56]
*Calendula officinalis* (Marigold)	Flavonoids, triterpenoids, carotenoids, phenolic acids	PUD, IBD, CRC	Anti-ulcer, anti-inflammatory, antioxidant, anticancer	[57]
*Aloe vera* (Aloe)	Anthraquinones, polysaccharides, phenolic compounds	PUD, IBD, CRC	Anti-ulcer, anti-inflammatory, immunomodulatory, anticancer	[58]
*Curcuma longa* (Turmeric)	Curcumin, curcuminoids, turmerones	PUD, IBD, CRC, LD	Anti-ulcer, anti-inflammatory, antioxidant, immunomodulatory, anticancer, hepatoprotective	[59]
*Zingiber officinale* (Ginger)	Gingerols, shogaols, zingerone	PUD, IBD, CRC, LD	Anti-ulcer, anti-inflammatory, antioxidant, immunomodulatory, anticancer, hepatoprotective	[60]
*Daucus carota* (Carrot)	Carotenoids, flavonoids, phenolic acids, polyacetylenes	GERD, PUD, IBS, IBD, CRC	Antacid, anti-inflammatory, antioxidant, anticancer, prebiotic	[61]
*Nerium oleander* (Oleander)	Cardiac glycosides, flavonoids, phenolic acids	PUD, IBD	Anti-ulcer, anti-inflammatory, immunomodulatory, cytoprotective	[45]
*Amaranthus viridis* (Amaranth)	Betalains, flavonoids, phenolic acids, saponins	PUD, IBD, CRC	Anti-ulcer, anti-inflammatory, antioxidant, anticancer, prebiotic	[62]
*Vaccinium myrtillus* (Bilberry)	Anthocyanins, flavonoids, phenolic acids	GERD, PUD, IBS, IBD, CRC	Antacid, anti-inflammatory, antioxidant, anticancer, anti-diarrheal	[47]
*Taraxacum officinale* (Dandelion)	Sesquiterpene lactones, flavonoids, phenolic acids, coumarins	GERD, PUD, IBS, IBD, LD	Antacid, anti-inflammatory, antibacterial, antispasmodic, hepatoprotective	[63]
*Punica granatum* (Pomegranate)	Anthocyanins, flavonoids, tannins, organic acids, and xanthonoids	CRC, UC, IBS, BUD, IBD	Anti-ulcer, anti-inflammatory, antioxidant, anticancer, Anxiolytics	[46]

GERD: gastroesophageal reflux disease; PUD: peptic ulcer disease; IBS: irritable bowel syndrome; IBD: inflammatory bowel disease; CRC: colorectal cancer; UC: ulcerative colitis.

### 3.2 PUD

PUD arises from acid peptic damage to the gastrointestinal tract, leading to the loss of the protective mucosal barrier. While peptic ulcers most commonly occur in the stomach or proximal duodenum, they can also be found in the esophagus or Meckel’s diverticulum [64]. The primary cause of PUD includes *Helicobacter pylori* infection and nonsteroidal anti-inflammatory medications (NSAIDs) [65]. The lifetime prevalence of PUD in the general population is estimated to be around 51%, with an annual incidence of 01–03% [64,65,66,67]. Due to the increasing frequency of antibiotic resistance, effective treatment of *H. pylori* infection has become an international concern [65]. The typical first-line therapy of PUD involved PPIs along with antibiotics, such as clarithromycin plus amoxicillin or metronidazole. However, the efficacy of this regimen in eradicating *H. pylori* has declined from over 90% two decades ago to less than 70% currently in many countries due to antibiotic resistance [65]. Table 2 highlights examples of Mediterranean plants, such as *T. vulgaris, Rosmarinus officinalis, Salvia officinalis, Origanum vulgare*, and *Mentha piperita*. These botanicals are well known for their therapeutic effects in managing PUD.

### 3.3 IBD

IBD is a chronic and recurrent gastrointestinal condition. The immune regulatory system, responsible for maintaining a delicate balance between tolerance and reactivity to gut microorganisms, becomes compromised in IBD. Common symptoms of IBD include diarrhea, stomach discomfort, weight loss, and fever. In addition, IBD can lead to severe physical and mental distress, significantly impacting both individuals and society, resulting in substantial health-care expenses [68]. The global prevalence of IBDs, including Crohn’s disease (CD) and UC, is increasing, notably in the Mediterranean area [69]. Approximately 2 million Europeans and 15 million North Americans are affected by IBD, with medical costs constituting a significant portion of health-care expenditures. Recent large-scale genome-wide association studies have identified more than 200 genetic loci linked to IBD, some of which are shared with other chronic autoimmune illnesses [70]. Clinically, anti-inflammatory agents (such as aminosalicylates and corticosteroids), immunosuppressants (such as azathioprine and methotrexate), and biologics (such as anti-TNF antibodies and anti-integrin antibodies) are the most commonly used drugs to treat IBD [68]. Many Mediterranean plants, including as *Curcuma longa, Zingiber officinale, Daucus carota, Amaranthus viridis*, and *Vaccinium myrtillus*, have prospective effects on IBD therapies. These plants contain active constituents that contribute to their therapeutic properties are listed in Table 2.

### 3.4 CRC

CRC remains a significant global health concern, with over 1.85 million cases and 850,000 deaths reported annually. As the third most common cause of cancer-related mortality worldwide, CRC poses substantial challenges for patients and healthcare systems. Notably, 25% of individuals initially diagnosed with localized disease will eventually develop metastases, while 20% of newly diagnosed CRC patients already present with metastatic disease. For patients with metastatic CRC that is not curable, treatment options primarily include cytotoxic chemotherapy and biologic therapy. The latter includes immunotherapy and antibodies treating cellular growth factors. Although extended lifespans remain relatively rare, there is a growing expectation of increased longevity. Genomic profiling makes it possible to select therapies, maximizing benefits for a broader population while minimizing exposure to the adverse effects of ineffective medications [71]. Despite advances in our understanding of CRC etiology, precursor lesions, and risk factors, a clear explanation for the recent rise in cancer cases among young people remains elusive. [72]. Further research is essential to unravel the underlying mechanisms driving this concerning trend. Table 2 includes examples of Mediterranean plants, such as *A. vera, Calendula officinalis, Gynostemma pentaphyllum, Ganoderma lucidum,* and *Cynodon dactylon*, as well as their active constituents that may have effects on CRC medication.

### 3.5 IBS

IBS is characterized by the abdominal discomfort during defecation or a change in bowel behavior [73]. Common symptoms include abdominal discomfort, bowel difficulty, and bloating, as well as the elimination of potentially dangerous symptoms such as unexpected weight loss, rectal bleeding, or a recent change in bowel function. Studies from Southeast Asia and the Middle East report a prevalence of 7.0%, that from North America, Europe, and Australasia report a prevalence of 11.8–14.0%, and that from South Europe, Africa, and South America report a prevalence of 15.0–21.0%. [74]. A meta-analysis of 56 global studies revealed that the prevalence of IBS tends to be slightly but considerably greater in women compared to men [75]. In general, IBS has a significant impact on the individual, affecting their quality of life and imposing significant societal and economic burdens [76]. Many Mediterranean plants such as *H. lupulus, Panax* spp., *Borago officinalis, Lavandula angustifolia,* and *Taraxacum officinale* are well known for their effects on IBS therapies. These plants contain active constituents that contribute to their therapeutic properties are listed in Table 2.

### 3.6 Liver digestive associated diseases

The liver, a vital organ, performs multiple essential functions such as blood filtration, bile production, nutrition metabolization, and blood volume regulation. However, the liver is susceptible to various disorders that impair its function and give rise to digestive complications. Liver malfunction inhibits normal nutrient digestion and absorption, potentially leading to gastrointestinal inflammation and bleeding. Some treatments of gastrointestinal LDs include medications (e.g., antibiotics, steroids, immunosuppressants, antivirals, chelating agents, anticancer drugs, and lifestyle modifications such as avoiding alcohol, losing weight, eating a healthy diet) [77,78,79]. Globally, LDs claim the lives of over 2 million people annually, and among these fatalities, cirrhosis accounts for approximately 1.16 million deaths. Alcohol and nonalcoholic fatty liver disease (NAFLD) are currently the main causes of cirrhosis in Western developed nations [80]. Liver damage is often associated with oxidative damage, an increase in tissue lipid peroxidation, aspartate aminotransferase (AST), alanine transaminase (ALT), alkaline phosphatase, total bilirubin, total protein, and cell necrosis [81,82]. Hepatic disease treatment is significantly affected by conventional therapies derived from natural sources. Active chemicals found in Mediterranean medicinal plants (Table 3) are utilized to treat digestive problems related to the liver. One such compound is silymarin, known for its antioxidant properties and its impact on enzyme systems involved in fibrosis and cirrhosis. Clinical trials have demonstrated that silymarin treatment significantly reduces liver-related mortality in patients with cirrhosis [83].

**Table 3:** Common LDs. The main Mediterranean plants’ active compounds used to treat LDs and their target mechanism of action

LD	Cause	Active compound	Plant source	Mechanism of action	References
Alcohol-related liver disease	Excessive alcohol consumption	Silymarin	Milk thistle (*Silybum marianum*)	Antioxidant, anti-inflammatory, antifibrotic and anti-apoptotic effects	[84]
Fatty liver disease	Excess fat accumulation	Curcumin	Turmeric (*Curcuma longa*)	Modulation of lipid metabolism, inflammation, oxidative stress, and insulin resistance	[85]
Hemochromatosis	Genetic, absorption too much iron	Phyllanthin	Bhumyamalaki (*Phyllanthus amarus*)	Chelation of iron and inhibition of iron absorption	[86]
Wilson disease	Genetic, preventing removal excess copper	Tetrathiomolybdate	Brassica spp.	Reduction of copper levels and inhibition of copper-dependent enzymes	[87]
Liver cancer	chronic viral infections, cirrhosis, exposure to toxins, and genetic factors	Betulinic acid	White birch (*Betula alba*)	Induction of apoptosis, inhibition of angiogenesis, and modulation of signaling pathways	[88]
Autoimmune hepatitis	Autoimmunity, attacks the liver cells	Glycyrrhizin	Licorice (*Glycyrrhiza glabra*)	Suppression of immune response, reduction of inflammation, and protection of hepatocytes	[89]
Primary sclerosing cholangitis	Bile ducts become inflamed and scarred	Silymarin	Milk thistle (*Silybum marianum*)	Antioxidant, anti-inflammatory, antifibrotic, and anti-apoptotic effects1, reduced hepatic TIMP-1/2	[90]
Primary biliary cholangitis	Autoimmunity, attacks the small bile ducts	Oleanolic acid	Olive (*Olea europaea*)	Anti-inflammatory, immunomodulatory, and hepatoprotective effects	[91]

Recent research has highlighted the potential of several natural compounds in mitigating liver damage. Among these compounds, resveratrol, glycyrrhetinic acid, phytolanthin, curcumin, and silymarin have demonstrated to possess both anti-inflammatory and antioxidant activities in animal models of LDs. These compounds show promise in reducing liver damage. Milk thistle silymarin, in particular, enhances hepatic glutathione and may contribute to the antioxidant defense of the liver [92,93]. In a randomized study involving 97 patients with histologically diagnosed mild, acute, and subacute LD caused by alcohol abuse, silymarin treatment for 4 our weeks resulted in a significantly greater improvement in liver function. This improvement was evidenced by a decrease in ALT and AST levels, compared with placebo [94]. Moreover, a double-blind controlled trial [94] demonstrated the efficacy of silymarin therapy in reducing elevated liver enzymes in patients with ALD, cirrhosis, and NAFLD. Silymarin has been significantly improved liver aminotransferases in patients with NAFLD without causing any particular negative effects, as shown in a randomized, double-blind, placebo-controlled trial [95]. Due to its anti-inflammatory properties, silymarin was found to downregulate the expression of NF-κB, IL-6, MMP-2, MMP-13, transforming growth factor beta-1, tumor-suppressor Krueppel-like factor, collagen α1 expression, and platelet-derived growth factor signaling in an alcoholic fatty liver model in rats [96,97].

Recent research has explored the therapeutic potential of curcumin in improving NAFLD, and it reduces the production of triglycerides (TG) [98] by inhibiting HMG-COA reductase [99]. In mice models, curcumin effectively reduces hepatic steatosis and lowers elevated hepatic TG [100]. When curcumin was supplemented to adult patients with metabolic syndrome, their body mass index values as well as their serum glucose levels, glycated hemoglobin, AST, ALT, TG, and total cholesterol were all below those of the placebo group [101]. Patients with NAFLD who received daily low-dose phospholipid curcumin supplementation for two months show a substantial decrease in their hepatic steatosis and enzyme levels when compared to placebo [102]. In addition, glycyrrhetinic acid showed to promote the growth of liver cells by binding to the epithelial growth factor receptor, enhancing the hepatic antioxidant defense, acting as an anti-inflammatory agent (Table 3), and activating the extracellular signal-regulated kinases (ERK2) pathway, along with stimulating DNA synthesis in liver cells [103,104]. 18β-glycyrrhetinic acid further reduces oxidative stress and the expression of inflammatory markers by downregulating of NF-κB and upregulating nuclear factor erythroid 2–related factor 2 (Nrf2) target genes. These effects were observed in both *in vitro* cel models and *in vivo* animal models with hepatic injury [105,106]. Furthermore, ethanolic extracts of *Phyllanthus amarus* exhibits strong hepatoprotective properties in both *in vitro* and *in vivo* settings. Recent studies have shed light on the potential benefits of Phyllanthus extract in the treatment of both acute and chronic hepatitis in children [38]. In addition, phytolanthin, a compound found in Phyllanthus, has demonstrated protective effects on rat livers exposed to galactosamine and carbon tetrachloride-induced cytotoxicity [107]. Another intriguing compound, resveratrol, plays a crucial role in safeguarding the liver following hepatocyte injury by modulating the expression of the nuclear transcription factors Nrf2 and NF-κB and downregulating the expression of HO-1 and iONS genes. Resveratrol effectively protects the liver following hepatocyte injury by reducing oxidative stress [108]. Furthermore, the Mediterranean foods, particularly plants, are thought to be rich in various kinds of chemical compounds that deserve clinical investigation to figure out their importance in treating digestive system-related liver illnesses.

## 4 MD importance in digestive diseases

Functional foods, enriched with specific minerals, vitamins, fatty acids, and dietary fibers, contain biologically active substances. These include phytochemicals, antioxidants, and probiotics, which have the potential to improve health and reduce disease risk [109,110,111]. Polyunsaturated fatty acids (found in olive oil and nuts) and antioxidative bioactive substances such as flavonoids, phytosterols, terpenes, and polyphenols exhibit anti-atherogenic and anti-inflammatory functions. Similarly, a perfect balance of micronutrients, including vitamins and minerals, which are rich in this diet, aids in the prevention of malnutrition and immunodeficiencies [112]. Concerning digestive disorders, high intakes of mono- and disaccharides, along with total lipids, for example, continuously increase the risk of developing IBD. Higher vegetable consumption may lower the incidence of UC, whereas higher fruit and/or dietary fiber consumption may offer protection against CD. Probiotics and prebiotics may influence gut microbiota and lessen the chance of IBD relapse. Previous studies have linked depression and emotional stress with the appearance of FGIDs such as functional dyspepsia and IBS [113,114]. Dietary patterns and the overall diet may be more relevant in illness risk than particular meals or nutrients [115]. Malnutrition impairs the clinical course of underlying disorders and is linked to poor clinical outcomes. Dietary control of gastrointestinal illness pathophysiology by manipulation of intestinal permeability and inflammation [116,117]. The individuals living in the Mediterranean Basin had underneath death rate with the prevalence of cardiovascular and cancer disorders than other populations, according to Ancel Benjamin. MD is marked by an excessive amount of grains, vegetables, fruit, olive oil, and tiny amounts of dairy products, pastry, sweets, and meat-based foods all simultaneously [118,119].

## 5 Mechanism of action of Mediterranean plants active compounds used to treat specific digestive diseases

**Figure 2:**
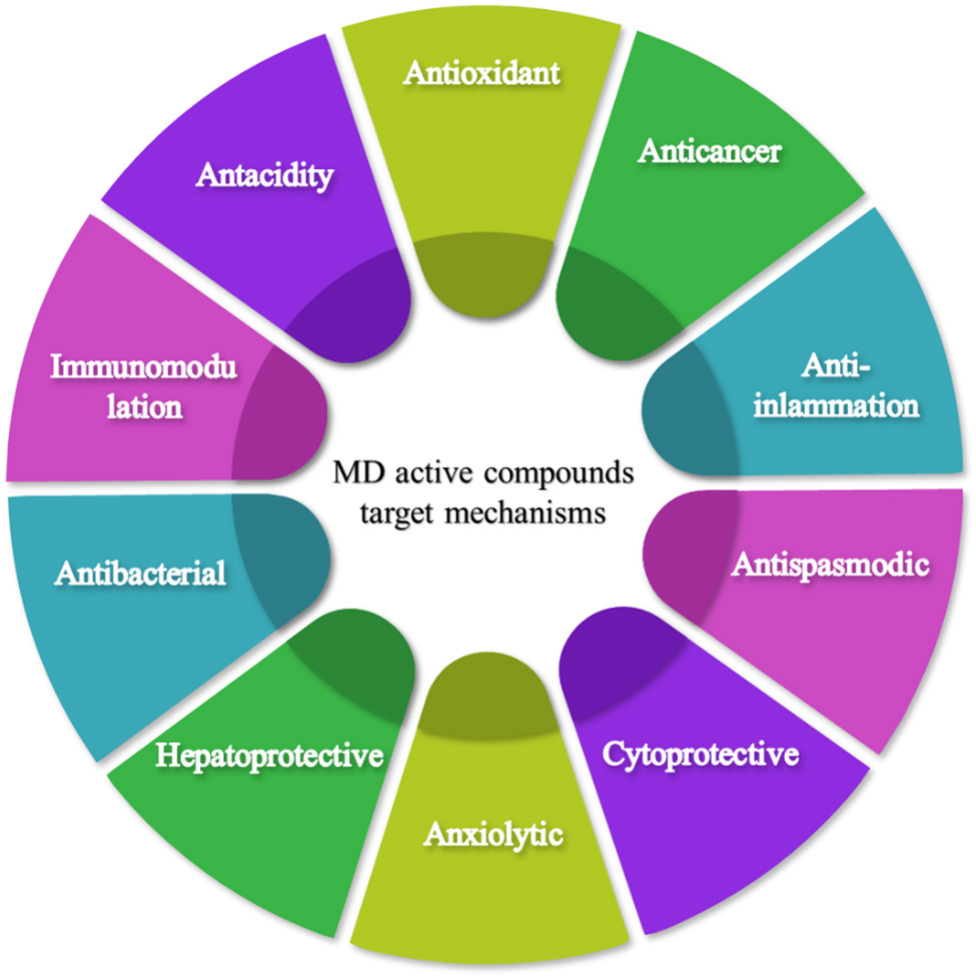
Mediterranean active compounds target mechanisms.

There is still much to learn about the exact mechanism of action by which the active chemicals in medicinal plants and MDs accomplish their therapeutic effects. Figure 2 shows the potential target mechanisms by which gastrointestinal tract diseases can be managed through medicinal herbs and their active phytocompounds.

### 5.1 Antacidity

The release of gastric acid by parietal cells in the stomach plays a crucial role in regulating the acidity of the digestive tract. However, an overabundance of acid, on the other hand, can cause a variety of digestive illnesses such as GERD, PUD, and nonulcer dyspepsia. Notably, peptic ulcers can result in severe complications such as excessive bleeding due to erosion of blood vessels [120]. To avoid excessive acid production, H2 antagonists, such as ranitidine, bind to H2 receptors, thereby inhibiting histamine binding and reducing acid output [121]. In addition, the hydrogen potassium ATPase enzyme, which is responsible for the final step of acid secretion into the stomach, is inhibited by PPI. Remarkably, PPI are prodrugs become active only after cleaving in the acidic secretory canaliculi of parietal cells by an acid. In the liver, P450 enzymes break down PPIs, with cytochrome P450 2C19 being the most common P450 enzyme involved in the breakdown of the different PPIs, albeit the specific P450 enzymes involved, although specific P450 enzymes may vary slightly [122,123]. Beyond conventional pharmaceutical approaches, antacid effects have been observed in phytochemicals. Flavonoids, alkaloids, tannins, and saponins are some phytochemicals that have been studied for their antacid characteristics. Notably, Mediterranean medicinal herbs such as *A. absinthium* and *H. lupulus* are two major Mediterranean medicinal herbs have been used as antacids [45,46,118,121]. However, while certain medicinal herbs have been demonstrated to have antacid characteristics, they should not replace standard medical therapy [124]. Table 2 provides an overview of 14 Mediterranean plants and their active constituents, which are well known for their antacid effects.

### 5.2 Anti-inflammation

**Figure 3:**
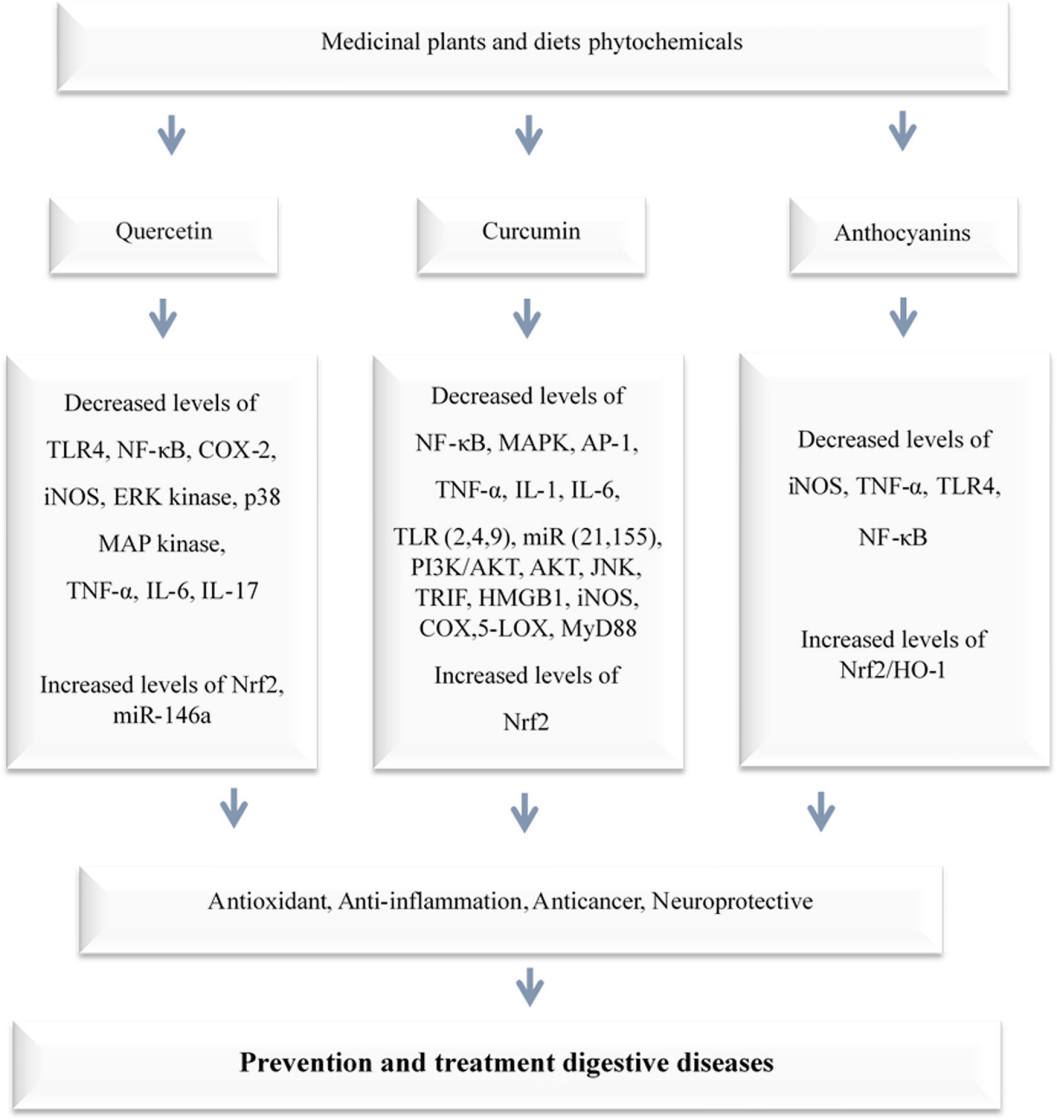
Major phytochemicals of Mediterranean medicinal plants and their antidigestive mechanisms. TLR: Toll-like receptor; NF-Kb: nuclear factor kappa B; COX: cyclooxygenase; iNOS: nitric oxide synthases; ERK2: extracellular signal-regulated kinases; MAPK: mitogen-activated protein; TNF-α: tumor necrosis factor alpha; IL: interleukin; Nrf2: nuclear factor erythroid 2–related factor 2; mIR: microRNA; AP-1: activator protein 1; PI3K: phosphoinositide 3-kinases; AKT: Protein kinase B; JNK: Jun N-terminal kinase; TRIF: TIR-domain-containing adapter-inducing interferon-β; HMGB1: high mobility group box 1; LOX: lysyl oxidase; MYD: myeloid differentiation primary response.

Inflammation is the body’s complicated biological respond to various stimuli such as germs, damaged cells, or irritants [125]. In the context of health, understanding inflammation and its modulation is crucial. Mediterranean medicinal plants rich in phytocompounds such as curcumin, resveratrol, anthocyanins, quercetin, and gingerol (Table 2) play a crucial role in reducing toll-like receptor (TLR) 4 receptor expression along with NF-κB transcription factor operation, hence minimizing the synthesis of downstream pro-inflammatory cytokines such as TNF-α, IL-6, and IL-1, as well as free radicals such as nitric oxide and reactive species (ROS). Furthermore, these phytochemicals stimulate the Nrf-2 signaling pathway, which falls oxidative stress [126]. Some plant chemicals can reduce inflammation by affecting miRNAs and genes that control it. For example, miR-155 and miR-21 are two miRNAs that increase inflammation by activating NF-κB and its target genes such as TNF-α, IL-6, and mitogen-activated proteins (MAPKs). Many plant chemicals mentioned earlier (Figure 3) can lower the levels of these miRNAs and reduce inflammation [125,127]. These plant chemicals can also change the activity of enzymes that modify the DNA and histones, such as histrone acetyl transferases and HDACs, and affect the expression of inflammatory genes [125]. Inflammation within the digestive system can cause problems like IBD, IBS, and GERD [125]. Following MD, which is rich in plant chemicals, can improve the symptoms and markers of inflammation in children and teens with mild to moderate IBD. After 12 weeks of therapy, the majority of the participants in a clinical investigation achieved recovery. Participants who followed the MD protocol had a substantial decrease in both clinical ratings (PCDAI and PUCAI) and most inflammatory markers (CRP, calprotectin, TNF-α, IL17, IL12, and IL13) when compared to those in the control group, with faster recovery in both PCDAI and CRP [128]. Mediterranean medicinal plants offer a natural arsenal against inflammation, providing valuable insights for therapeutic strategies.

#### 5.2.1 Quercetin

Quercetin is a flavonoid abundant in various dietary sources, including citrus fruits, apples, onions, red grapes, and tea [125]. This phenolic molecule exhibits a remarkable array of health-promoting issues and serves as anti-inflammatory, antioxidant, chemopreventive, and neuroprotective [129]. In macrophages and human PBMCs, quercetin decreases the production of TLR4 and hinders NF-κB trafficking to the nucleus, which alleviates the inflammatory response [125]. It also suppresses cyclooxygenase (COX)-2 and nitric oxide synthases (iNOS) gene expression *in vitro* and greatly lowers the generation of proinflammatory cytokines in LPS-activated macrophages via the MAP kinases and NF-κB pathway [130]. In LPS-treated RAW 264.7 macrophages, quercetin inhibits NF-κB activation via regulating the NF-κB/IB complex and reduces phosphorylated ERK kinase and p38 MAP kinase [130]. It has been observed that quercetin and its metabolite can also suppress the NF-κB pathway indirectly by activating the Nrf2 signaling cascade [131]. Furthermore, quercetin has been demonstrated to upregulate miR-146a, which is followed by a reduction in NF-κB, TNF-α, IL-6, and IL-17 levels. The quercetin content of Mediterranean plants may have cytoprotective properties in digestive diseases such as IBD, IBS, and GERD [125].

#### 5.2.2 Curcumin

Curcumin, a polyphenol derived from the ginger plant *C. longa* [132,133] plays a pivotal role in managing various inflammatory conditions, including rheumatoid arthritis, IBD, nephropathies, and certain malignancies [125]. Curcumin has a strong anti-inflammatory effect, which is attributed to its regulation of the TLR4 and MyD88 pathways in macrophages, and it effectively prevents NF-κB activation [134,135]. In addition, curcumin suppresses MAPK and AP-1 transcription factor activation along with inhibiting IB-phosphorylation and degradation [136,137]. These antagonistic effects on TLR4 signaling pathways and downstream mediators are followed by suppression of proinflammatory cytokines such as TNF-α, IL-1, and IL-6 [125]. A research indicates that curcumin pretreatment protects against T cell-mediated hepatitis in mice. The strong impact of curcumin may be attributed in part to decreasing the expression levels of TLR2, TLR4, and TLR9 in the liver [138]. Moreover, curcumin was found to downregulated miR-155 levels *in vivo* and *in vitro* following LPS stimulation via depleting the phosphoinositide 3-kinase PI3K/AKT pathway [139]. Regular consumption of curcumin reduces the expression of miR-21 and miR-155 in clinical investigations. Consequently, this was followed by a reduction of the proliferative kinases AKT and JNK, as well as the transcription factor AP-1, which reduced inflammation by decreasing NF-κB activation, TNF-α, and IL-6 production [140,141]. Curcumin is typically taken three times per day at doses ranging between 400 and 600 mg. Remarkably, it exhibits no adverse effects on the kidney or liver when taken up to 12 g/day [142,143]. However, extremely high dosages may lead to stomach distress and gastric ulcers [144]. Hence, curcumin, which is a key ingredient in Mediterranean traditional medicine, may be a safer compared with NSAIDs for inflammation. A study indicates that curcumin can decrease the development of TLR2, TLR4, and HMGB1 in rats suffering from fibrogenesis expression of ligand molecules, suggesting that curcumin’s hepatoprotective mechanism may be attributed to its antioxidant activity [145,146]. In addition, curcumin may lower other ROS-producing enzymes such as iNOS, the COX system, and arachidonate 5-lipoxygenase, as well as reducing the action of numerous pyoderma gangrenosums [144].

#### 5.2.3 Anthocyanins

Anthocyanins, flavonoids present in many fruits and vegetables, particularly in the Mediterranean area, represent a part of the traditional diet [147]. They may aid in the prevention or treatment of digestive illnesses such as IBD, UC, CD, and CRC. Anthocyanin has recently been shown to be beneficial in the treatment of fatty liver and inflammation (125). Anthocyanins, in particular, reduce proinflammatory mediators like as iNOS and TNF-α by lowering TLR4 expression and inactivating NF-κB [148]. They also help to protect against oxidative stress by stimulating the Nrf2/HO-1 signaling pathway [149]. Anthocyanins have been shown to inhibit proinflammatory TNF-α signaling and gene expression in murine livers [150]. A clinical investigation, including individuals with UC for example, found that supplementation with anthocyanin-rich bilberry extract improved patients’ activity and quality of life. A recent study shed the light on the health-promoting effects of anthocyanins derived from purple sweet potato extracts. It decreased colonic inflammation and inhibited the development of carcinogenesis in a rat model of colitis-associated CRC. Furthermore, a meta-analysis of epidemiological data supports the potential benefits of anthocyanin consumption. Increased intake of anthocyanin was associated with a decreased risk of CRC [147,151].

### 5.3 Antibacterial

Bacterial infections of the gastrointestinal system are most commonly caused by bacteria or bacterial toxins of contaminated food or drink. Dental caries, periodontal disease, shigellosis, salmonellosis, and typhoid fever exemplify bacterial disorders of the digestive system [152,153,154]. However, plants such as *A. absinthium*, *H. lupulus*, *F. vulgare*, *T. vulgaris*, *T. officinale,* and their derived metabolites of different Mediterranean habit (Table 2) inhibit damaging factors of pathogenic microorganisms. There have been reports of phytochemical components that provide a fascinating defense mechanism against predation by a variety of Gram-positive and Gram-negative bacteria, along with antibiotic-resistant bacteria. These constituents include tannins, flavonoids, alkaloids, glycosides, cyanogenetic glycosides, reducing sugar, and several other aromatic compounds [155,156]. Reports suggest that flavonoids and polyphenolic chemicals may have antibacterial activity because of their capacity to bind to bacterial cell walls and impede microbial development [157]. In addition, antivirulence phytochemical research has mostly focused on *in vitro* quorum quenching with their antibiofilm characteristics. Notably, myristic acid, which reduces virulence *in vitro*, paradoxically acts as a signal molecule that increases *Pseudomonas aeruginosa* pathogenicity in a dermonecrotic animal model. Moreover, certain mouse models have revealed that type three secretion systems play a pivotal role as virulence determinants [158]. Anthocyanins, abundant in various foods, may help the digestive tract by reducing oxidative stress, alleviating inflammation, and modulating gut flora [159].

### 5.4 Antispasmodic

Medicines with antispasmodic properties are frequently used to lessen excessive smooth muscle contractility, which often leads to stomach pain and cramping associated with various gastrointestinal, biliary, or genitourinary tract conditions [160]. A significant portion of the population experience conditions such as IBS, biliary colic brought on by gallstones, gastritis, colitis, pancreatitis, or dysmenorrhea, all of which commonly require antispasmodic medication to reduce symptoms [161,162,163,164]. Antispasmodic drugs are also employed to alleviate the pain during medical procedures such as colonoscopy [165]. Multiple Mediterranean medicinal herbs, including *Zanthoxylum armatum*, *Matricaria chamomilla*, *F. vulgare*, *Pycnocycla spinosa*, *Atropa belladonna*, *L. angustifolia*, *Mentha pulegium*, *Glycyrrhiza ularensis*, *Anethum graveolens*, and *Origanum majorana* contribute to symptom relief through their antispasmodic properties [161]. Phytochemicals have been demonstrated to have antispasmodic characteristics, particularly secondary metabolites such as terpenes, phenolics, and alkaloids, are responsible for these plants’ pharmacological actions [162].

### 5.5 Cytoprotective

The capacity of cells to endure damaging stimuli while maintaining structural and functional integrity is referred to cytoprotecting. Cytoprotective drugs prevent or minimize damage to the gastrointestinal mucosa caused by different variables such as acid, pepsin, and *H. pylori* in the setting of digestive illnesses [166]. Various Mediterranean medicinal herbs, including *Olea europaea*, *Rosmarinus officinalis*, *T. vulgaris*, *O. vulgare*, and *Salvia officinalis*, have cytoprotective action [167]. Phytochemicals including curcumin, resveratrol, quercetin, and gingerol that target TLR4/NF-κB-mediated inflammatory mechanisms have cytoprotective activities [167].

### 5.6 Anxiolytics

Anxiolytics, a class of medications, exert their effects by targeting the gamma-aminobutyric acid (GABA) receptor. This receptor regulates chloride ion entry into neurons, leading to neuronal hyperpolarization [168]. However, the precise mechanism of action of anxiolytics is yet unknown [169]. Several medicinal herbs have been explored for their anxiolytic properties including pomegranate, lavender, hops, maypop, lemon balm, valerian, and peppermint [170]. Pomegranate contains various phytochemicals, such as anthocyanins, flavonoids, tannins, organic acids, and xanthonoids, and have demonstrated anxiolytic properties [171]. Phytochemicals in pomegranate likely exert their effects through multiple ways, including inhibition of GABAergic receptors, modulation of N-methyl-D-aspartate and CaMKII/CREB pathways, reduction of oxidative stress via TLR4 and iNOS inhibition, control of cytokines and NF-kB generation, and activation of Nrf2 and AMPK. While anxiolytics offer relief, they may come with undesirable side effects, including addiction, depression, suicidal tendencies, seizures, sexual dysfunction, and headaches [172]. Exploring the anxiolytic potential of medicinal herbs provides valuable insights for managing anxiety while minimizing adverse effects associated with conventional medications.

### 5.7 Antioxidant

Antioxidants are molecules that have the potential to neutralize free radicals and prevent them from causing cell injury. In digestive diseases, oxidative stress can trigger inflammation and mucosal damage [173]. According to scientific studies, antioxidant properties are the key mechanism for phytochemicals to alleviate various disease pathways by increasing the antioxidant defense mechanisms of cells, scavenging free radicals, decreasing lipid peroxidation, boosting anti-inflammatory potential, and further protecting hepatic cell damage [84]. Terpenoids (such as monoterpenes and carotenoids) and polyphenols (such as quercetin and other flavonoids) are significant phytochemicals having antioxidant properties. In this context, lavender, hops, maypop, lemon balm, valerian, and peppermint are among the therapeutic herbs used to treat antioxidant-related illnesses [174]. In addition, bioactive phytochemicals from *Hypericum* species exhibit both anti-inflammatory and antioxidant properties [175]. Numerous antioxidant compounds have been found to be abundant in *Rosmarinus officinalis*, *Z. officinale*, *F. vulgare*, *C. longa*, and fragrans. Because these foods are antioxidant rich, regular consumption of these foods offers additional health benefits [176]. Quercetin is a strong antioxidant that suppresses the generation of lipid peroxides and lysosomal enzymes like the acid phosphatase and cathepsin D [177,178]. On the other hand, curcumin reduces oxidative damage during inflammation by stimulating the Nrf2-Keap1 pathway and enhancing antioxidant enzyme efficiency [179].

### 5.8 Immunomodulatory

Immunomodulators are chemicals that alter or control the immune system to improve the body’s response to a medical condition and diseases. Immunomodulators can help regulate symptoms and preserve immune system homeostasis in the context of digestive diseases [180]. Several Mediterranean medicinal herbs, including *Echinacea purpurea*, *Panax ginseng*, *C. longa*, and *Allium sativum*, are used to treat immunomodulatory-related disorders [181]. Medicinal plants and isolated compounds with immunomodulatory properties are prospective treatments for digestive diseases and viral infections such as COVID-19. Polysaccharides, terpenoids, flavonoids, alkaloids, glycosides, and lactones are plant phytochemicals that play a crucial role in immunomodulation [182].

### 5.9 Anticancer

The intricacies of cancer pathobiology are strongly linked to the primary challenges in developing target-specific anticancer medications. Autophagy, alongside apoptosis, plays a critical role in cellular mechanisms related to cancer genesis and treatment. However, due to the abnormalities in signaling pathways, notably apoptosis, many tumor types developed resistance against treatment. Autophagy might be investigated as another cell fate mechanism for the exploration of target-specific antitumor medicines [183]. Growing evidence suggests that phytochemicals, which alter various signaling targets, including both autophagy and apoptosis, exhibit anticancer outcome [184]. Phytocompounds anticancer actions were shown to be selective and specific to cancer cells, involving the control of autophagy and apoptosis [183]. Numerous studies have documented the protective effects of curcumin against UC, CD, pancreatic, and colorectal malignancies [185]. In addition, researchers have explored phytochemicals derived from Mediterranean plants across a range of cancer cell lines to uncover their critical biological pathways in cancer genesis and control (Table 4) [183].

**Table 4:** Phytochemical compounds from Mediterranean plants and their *in vitro* biological regulatory pathways in the genesis and control of cancer

Active compound	Cell line	Biological pathway
Resveratol	Human colon carcinoma	Activate apoptosis-3 and 8/FADD
Eriocalyxin	Human pancreatic cancer	Activate caspase 8 and 9 inhibit caspases 3 and 7
Apigenin	CRD	Activate NAG-1, p53, cleave caspase 3
Allicin	Human gastric cancer	Inhibit p38 expression and cleave caspase 3
Evodiamine	Human gastric cancer	Activate beclin-2, Bax, downregulate Bcl-2
Gingerol	Human colon cancer	Inhibit JNK, ERK1-2, P38 MAPK
Toxicarioside O	CRD	Inhibit Akt/Mtor
Oleanolic acid	Human pancreatic	Modulate JNK, mTOR pathway
Oridonin	Human hepatocellular carcinoma	Activation caspase-3 downregulate Bcl-2 and upregulate Bax
Thymoquinone	Oral cancer	Increase expression of LC3-II, Bax expression
Tetrandrine	Hepatocellular carcinoma	inhibit Wnt/Beta-catenin
Quinacrine	Human colon cancer	Activation p53, p21, inhibit topoisomerase
Chloroquine	Pancreatic cancer	Decrease the level of O_2_
Isorhamnetin	Colon cancer	Increase ROS
Benzylisothiocyanates	Pancreatic cancer	Decrease phosphorylation of PI3K/Akt/FOXO1/PDK1/mTOR/FOXO3a
Kaempferol	CRD	Generates ROS and p53 signal
Triptolide	Human pancreatic	Inhibit Akt-mTOR-p70S6K

NAG-1: nonsteroidal anti-inflammatory drug-activated gene 1; Bax: Bcl-2-associated X protein; JNK: Jun *N*-terminal kinase; ERK2: extracellular signal-regulated kinases; MAPK: mitogen-activated protein; AKT: protein kinase B; mTOR: mammalian target of rapamycin; JNK: Jun *N*-terminal kinase; LC3-II: phosphatidylethanolamine conjugate II; ROS: reactive species; PI3K: phosphoinositide 3-kinases; PI3K/Akt/FOXO1/PDK1/mTOR/FOXO3a; signaling pathway, p70S6K: p70 ribosomal S6 kinase.

### 5.10 Hepatoprotective

Based on experimental validation, natural bioactive substances derived from plants are promising options for predicting and alleviating hepatotoxic effects and chronic problems [84]. Several in silico investigations and molecular networking substances have also highlighted active phytochemicals derived from natural sources as potential hepatoprotective agents [84]. Plant products contain a range of phytochemicals that exhibit the hepatoprotective effect against CCl4-induced toxicity by downregulating liver marker enzymes and activating the antioxidative capability of the liver cells, resulting in liver repair [88]. Despite the fact that these drugs show significant hepatoprotective benefits in animal and cell culture models, a lack of clinical research continues to be a barrier to their official recognition. Therefore, controlled clinical studies are required to establish the therapeutic effectiveness of possibly hepatoprotective substances. Understanding the fundamentals of phytochemical hepatoprotective action might guide future medication development and aid in the prevention of clinical trial failure. Furthermore, the development of innovative delivery methods that improve the bioavailability of weakly water-soluble drugs may improve the findings that have previously been obtained. Most significantly, published data indicate that phytochemicals modulate distinct signaling pathways to varying degrees, highlighting the necessity for the use of mixtures of numerous hepatoprotective substances in both experimental research and clinical trials [186]. Many plants, such as the roots of *T. officinale* and the leaves of *Mentha longifolia*, are utilized in various herbal formulations as hepatoprotectives. It has been discovered that curcumin prevents the hepatitis C virus from entering by changing the fluidity of the membrane and the way the virus binds and fuses [185]. In addition, total saponins from *Panax notoginseng* have been shown to have a promising effect on fatty LD [187]. Dietary polyphenols and their metabolites have been also found to be crucial for preserving the microbial balance and overall health of the gut [185].

## 6 Challenges of MD for health and well-being

The MD has been demonstrated to be an appropriate dietary regimen that may lower the risk of noncommunicable diseases [188]. Promoting the MD as a healthy dietary pattern, on the other hand, offers obstacles that require the involvement of all levels of society [188]. One difficulty is the lack of a uniform definition and scoring system for the MD [189]. Another problem is the effect of nutritional transition, which encourages the move of traditional foods to Westernized diets, complicating adherence to the MD [189]. Despite its growing global appeal, adherence to the MD model is declining due to a variety of variables such as lifestyle changes, food globalization, economic, and sociocultural considerations [190]. These alterations endanger the maintenance and transfer of the Mediterranean food legacy to future generations [190]. Interestingly, middle-aged and older Hispanic or Latino people who adhere to the MD experience improved cognitive function and reduced 7-year learning and memory loss. MD that is culturally adjusted may minimize the risk of cognitive decline and Alzheimer’s disease [191]. More cross-disciplinary research on the environmental, economic, sociocultural, and sustainability components of the MD is expected to be extremely important [190]. MD containing plenty of plant foods with limited processing has been associated with increased life expectancy and a decreased risk of developing a number of chronic diseases [192]. Moreover, a Mediterranean-style diet may reduce the prevalence of gestational diabetes mellitus in pregnant women with metabolic risk factors [193]. A Mediterranean-style diet has also been shown to reduce the likelihood of GERD symptoms [194]. Unfortunately, as life and the drug industry have advanced, this knowledge has become steadily less communicated and is on the verge of extinction, necessitating the need to preserve and integrate traditional medicine into the current health system through ethnobotany and ethnopharmacology [195]. As the diet is contributing mainly in obesity, it raised the risk of a variety of benign digestive disorders. Obesity-induced mechanical and humoral variables are implicated in the development of esophageal illnesses, whereas obesity-induced proinflammatory and inflammatory cytokines appear to be involved in the pathophysiology of other digestive disorders. Furthermore, excess weight and obesity increase free fatty acid, TNF-α, and resistin levels while decreasing adiponectin. This causes insulin resistance and changes in the IGF-1 pathway, as well as inhibiting apoptosis and increasing cell proliferation on target cells. Obesity elevates free fatty acids and modifies adipocytokines. This metabolic change results in metabolic syndrome, which includes insulin resistance, dyslipidemia, and hypertension. In addition, metabolic changes and metabolic syndrome have a role in both benign and malignant digestive disorders. Obesity’s mechanical impact may lead to esophageal disease and other digestive disorders [196].

## 7 Conclusion

Disorders affecting the digestive system have a substantial impact on global health, resulting in elevated rates of illness and mortality. These challenges are particularly pronounced in rural areas where knowledge about sanitation and disease prevention is limited. In less developed countries, plant-based therapies frequently serve as the primary approach for preventing and treating gastrointestinal diseases. Numerous studies have validated the traditional use of these therapies by investigating their pharmacological effects on various diets and plants. These effects include reducing gas, relieving spasms, slowing intestinal transit, influencing gut motility, stimulating absorption, or decreasing electrolyte secretion. The MD, renowned for its rich variety of plant-based foods, is acknowledged as a dietary pattern that promotes overall health and well-being. This diet incorporates foods high in polyphenols, such as olive oil, walnuts, vegetables, fruits, legumes, wild edible plants, and whole grains, all of which are known to provide digestive benefits. Phytochemicals derived from plants and diet, particularly polyphenols, can function independently or synergistically to mitigate digestive system complications. Present scientific assessments of plant-based products primarily aim to validate and identify active components in extracts and other preparations. However, certain herbal preparations or active ingredients can cause severe side effects under specific conditions. As a result, it is crucial to establish a scientific foundation for their activity and to better evaluate the quality, effectiveness, and safety of traditionally used diets and medicinal plant-based preparations. Well-designed clinical trials are required to verify herbal medicines and offer the proof that’s needed to justify their efficacy. The safety and tolerability of traditional and herbal medications used to treat problems of the digestive system have not been well studied in clinical trials, but those that have been performed have generally shown minimal adverse effects. To create alternative treatments for digestive disorders, more research on traditional plant-based remedies is necessary given the promising results of previous studies.

## References

[1] Wang Y, Huang Y, Chase RC, Li T, Ramai D, Li S, et al. Global burden of digestive diseases: A systematic analysis of the global burden of diseases study, 1990-2019. Gastroenterology. 2023 Jun;165(3):773–83. 10.1053/j.gastro.2023.05.050 37302558

[2] Cao X, Zolnikova O, Maslennikov R, Reshetova M, Poluektova E, Bogacheva A, et al. Low short-chain-fatty-acid-producing activity of the gut microbiota is associated with hypercholesterolemia and liver fibrosis in patients with metabolic-associated (Non-Alcoholic) fatty liver disease. Gastrointest Disord. 2023 Oct 30;5(4):464–73.

[3] Kliegman RM, Toth H, Bordini BJ, Basel D, editors. Nelson pediatric symptom-based diagnosis. Nelson: Elsevier Health Sciences; 2022 Jan.

[4] Hazel K, O’Connor A. Emerging treatments for inflammatory bowel disease. Ther Adv Chronic Dis. 2020 Feb;11:2040622319899297. 10.1177/2040622319899297 PMC7003169 32076497

[5] Cai Z, Wang S, Li J. Treatment of inflammatory bowel disease: a comprehensive review. Front Med. 2021 Dec;8:765474. 10.3389/fmed.2021.765474 PMC8720971 34988090

[6] Atanasov AG, Zotchev SB, Dirsch VM, Supuran CT. International natural product sciences taskforce. Nat Rev Drug Discov. 2021;20:200–16. 10.1038/s41573-020-00114-z PMC7841765 33510482

[7] Cuvelier ME. Antioxidative activity and phenolic composition of pilot-plant and commercial extracts of sage and rosemary. JAOCS. 2002;162:981–7.

[8] El-Darier SM, El-Mogaspi FM. Ethnobotany and relative importance of some endemic plant species at El-Jabal El-Akhdar Region (Libya). World J Agric Sci. 2009;5(3):353–60.

[9] Ezzoubi Y, Bousta D, Farah A. A Phytopharmacological review of a Mediterranean plant: Lavandula stoechas L. Clin Phytoscience. 2020 Dec;6:1–9.

[10] Grigoriadou K, Krigas N, Lazari D, Maloupa E. Sustainable use of mediterranean medicinal-aromatic plants. In Feed additives. Cambridge, Massachusetts: Academic Press; 2020. p. 57–74.

[11] Cheema HS, Prakash O, Pal A, Khan F, Bawankule DU, Darokar MP. Glabridin induces oxidative stress mediated apoptosis like cell death of malaria parasite Plasmodium falciparum. Parasitol Int. 2014 Apr;63(2):349–58. 10.1016/j.parint.2013.12.005 24361284

[12] Kmail A, Mansour B, Hanaisheh R, Omar G, Jaradat N, Said O, et al. Modulatory effects of leave and fruit extracts of ficus sycomorus on cytostatic and inflammatory mediators in monocultures and Co-cultures of human Keratinocyte (HaCat) and human Monocyte (THP-1) cell lines. EJMP. 2022;33(9):1–14.

[13] Granato D, Barba FJ, Bursać Kovačević D, Lorenzo JM, Cruz AG, Putnik P. Functional foods: Product development, technological trends, efficacy testing, and safety. Annu Rev Food Sci Technol. 2020 Mar;11:93–118. 10.1146/annurev-food-032519-051708 31905019

[14] Jakubczyk D, Leszczyńska K, Górska S. The effectiveness of probiotics in the treatment of inflammatory bowel disease (IBD) – a critical review. Nutrients. 2020 Jul;12(7):1973. 10.3390/nu12071973 PMC7400428 32630805

[15] Mack DR. Probiotics in inflammatory bowel diseases and associated conditions. Nutrients. 2011 Feb;3(2):245–64. 10.3390/nu3020245 PMC3257670 22254095

[16] Brownawell AM, Caers W, Gibson GR, Kendall CW, Lewis KD, Ringel Y, et al. Prebiotics and the health benefits of fiber: current regulatory status, future research, and goals. J Nutr. 2012 May;142(5):962–74. 10.3945/jn.112.158147 22457389

[17] Carreras-Torres R, Ibanez-Sanz G, Obon-Santacana M, Duell EJ, Moreno V. Identifying environmental risk factors for inflammatory bowel diseases: a Mendelian randomization study. Sci Rep. 2020;10(1):19273. 10.1038/s41598-020-76361-2 PMC7648100 33159156

[18] Blanton C, He Z, Gottschall-Pass KT, Sweeney MI. Probiotics blunt the anti-hypertensive effect of blueberry feeding in hypertensive rats without altering hippuric acid production. PLoS One. 2015 Nov;10(11):e0142036. 10.1371/journal.pone.0142036 PMC4636313 26544724

[19] Odes HS, Madar Z. A double-blind trial of a celandin, aloevera and psyllium laxative preparation in adult patients with constipation. Digestion. 1991 Feb;49(2):65–71. 10.1159/000200705 1800188

[20] Panahi Y, Khedmat H, Valizadegan G, Mohtashami R, Sahebkar A. Efficacy and safety of Aloe vera syrup for the treatment of gastroesophageal reflux disease: a pilot randomized positive-controlled trial. J Tradit Chin Med. 2015 Dec;35(6):632–6. 10.1016/s0254-6272(15)30151-5 26742306

[21] Menzel J, Jabakhanji A, Biemann R, Mai K, Abraham K, Weikert C. Systematic review and meta-analysis of the associations of vegan and vegetarian diets with inflammatory biomarkers. Sci Rep. 2020 Dec;10(1):21736. 10.1038/s41598-020-78426-8 PMC7730154 33303765

[22] Tsoupras A, Lordan R, Zabetakis I. Inflammation, not cholesterol, is a cause of chronic disease. Nutrients. 2018 May;10(5):604. 10.3390/nu10050604 PMC5986484 29757226

[23] Elmaliklis IN, Liveri A, Ntelis B, Paraskeva K, Goulis I, Koutelidakis AE. Increased functional foods’ consumption and Mediterranean diet adherence may have a protective effect in the appearance of gastrointestinal diseases: a case–control study. Medicines. 2019 Apr;6(2):50. 10.3390/medicines6020050 PMC6631641 30970582

[24] Park JY, Nicolas G, Freisling H, Biessy C, Scalbert A, Romieu I, et al. Comparison of standardised dietary folate intake across ten countries participating in the European Prospective Investigation into Cancer and Nutrition. Br J Nutr. 2012 Aug;108(3):552–69. 10.1017/S0007114511005733 22040523

[25] Merra G, Noce A, Marrone G, Cintoni M, Tarsitano MG, Capacci A, et al. Influence of mediterranean diet on human gut microbiota. Kompass Nutr Dietetics. 2022 Apr;2(1):19–25. 10.3390/nu13010007 PMC7822000 33375042

[26] Hidalgo-Mora JJ, García-Vigara A, Sánchez-Sánchez ML, García-Pérez MÁ, Tarín J, Cano A. The Mediterranean diet: A historical perspective on food for health. Maturitas. 2020 Feb;132:65–9. 10.1016/j.maturitas.2019.12.002 31883665

[27] Saad B, Dakwar S, Said O, Abu-Hijleh G, Battah FA, Kmeel A, et al. Evaluation of medicinal plant hepatotoxicity in co-cultures of hepatocytes and monocytes. Evidence-Based Complementary Altern Med. 2006 Mar;3:93–8. 10.1093/ecam/nel002 PMC1375247 16550229

[28] Kokkini S, Karousou R, Dardioti A, Krigas N, Lanaras T. Autumn essential oils of Greek oregano. Phytochemistry. 1997 Mar;44(5):883–6.

[29] Lis A, Kowal M, Kończak J. Chemical Composition Variability of the Herb Essential Oil in the Ontogenesis of Artemisia campestris subsp. campestris. Nat Product Commun. 2015 Oct;10(10):1934578X1501001032. 26669121

[30] Saad B. A review of the anti-obesity effects of wild edible plants in the mediterranean diet and their active compounds: from traditional uses to action mechanisms and therapeutic targets. Int J Mol Sci. 2023 Aug;24(16):12641. 10.3390/ijms241612641 PMC10454857 37628822

[31] Sitarek P, Merecz-Sadowska A, Kowalczyk T, Wieczfinska J, Zajdel R, Śliwiński T. Potential synergistic action of bioactive compounds from plant extracts against skin infecting microorganisms. Int J Mol Sci. 2020 Jul;21(14):5105. 10.3390/ijms21145105 PMC7403983 32707732

[32] Goh BH, Mocan A, Xiao J, Mah SH, Yap WH. Targeting human inflammatory skin diseases with natural products: exploring potential mechanisms and regulatory pathways. Front Pharmacology. 2021 Nov;12:791151. 10.3389/fphar.2021.791151 PMC8634468 34867426

[33] Delgado A, Gonçalves S, Romano A. Mediterranean diet: the role of phenolic compounds from aromatic plant foods. Foods. 2023 Feb;12(4):840. 10.3390/foods12040840 PMC9957056 36832914

[34] Arnesen EK, Thorisdottir B, Bärebring L, Söderlund F, Nwaru BI, Spielau U, et al. Nuts and seeds consumption and risk of cardiovascular disease, type 2 diabetes and their risk factors: a systematic review and meta-analysis. Food Nutr Res. 2023;67:1–33. 10.29219/fnr.v67.8961 PMC9930735 36816545

[35] Detopoulou P, Demopoulos CA, Antonopoulou S. Micronutrients, phytochemicals and mediterranean diet: A potential protective role against COVID-19 through modulation of PAF actions and metabolism. Nutrients. 2021 Jan;13(2):462. 10.3390/nu13020462 PMC7911163 33573169

[36] Arnoni Y, Berry EM. On the origins and evolution of the Mediterranean diet. In The Mediterranean Diet. London: Academic Press; 2015 Jan. p. 3–11.

[37] Sperber AD, Bangdiwala SI, Drossman DA, Ghoshal UC, Simren M, Tack J, et al. Worldwide prevalence and burden of functional gastrointestinal disorders, results of Rome Foundation Global Study. Gastroenterology. 2021 Jan;160(1):99–114. 10.1053/j.gastro.2020.04.014 32294476

[38] Peery AF, Crockett SD, Murphy CC, Jensen ET, Kim HP, Egberg MD, et al. Burden and cost of gastrointestinal, liver, and pancreatic diseases in the United States: update 2021. Gastroenterology. 2022 Feb;162(2):621–44. 10.1053/j.gastro.2021.10.017 PMC10756322 34678215

[39] Sepanlou SG, Malekzadeh F, Delavari F, Naghavi M, Forouzanfar MH, Moradi-Lakeh M, et al. Burden of gastrointestinal and liver diseases in Middle East and North Africa: results of global burden of diseases study from 1990 to 2010. Middle East J Digestive Dis. 2015 Oct;7(4):201. PMC4655840 26609348

[40] Baklola M, Terra M, Badr A, Fahmy FM, Elshabrawy E, Hawas Y, et al. Prevalence of gastro-oesophageal reflux disease, and its associated risk factors among medical students: a nation-based cross-sectional study. BMC Gastroenterol. 2023 Aug;23(1):269. 10.1186/s12876-023-02899-w PMC10405472 37550667

[41] Shaheen NJ, Hansen RA, Morgan DR, Gangarosa LM, Ringel Y, Thiny MT, et al. The burden of gastrointestinal and liver diseases, 2006. J Am Coll Gastroenterol ACG. 2006 Sep;101(9):2128–38. 10.1111/j.1572-0241.2006.00723.x 16848807

[42] Fass R, Ofman JJ, Gralnek IM, Johnson C, Camargo E, Sampliner RE, et al. Clinical and economic assessment of the omeprazole test in patients with symptoms suggestive of gastroesophageal reflux disease. Arch Intern Med. 1999 Oct;159(18):2161–8. 10.1001/archinte.159.18.2161 10527293

[43] Fass O, Sampliner C, Wendel F. The omeprazole test is as sensitive as 24‐h oesophageal pH monitoring in diagnosing gastro‐oesophageal reflux disease in symptomatic patients with erosive oesophagitis. Aliment Pharmacol Ther. 2000 Apr;14(4):389–96. 10.1046/j.1365-2036.2000.00733.x 10759617

[44] Roark R, Sydor M, Chatila AT, Umar S, De La Guerra R, Bilal M, et al. Management of gastroesophageal reflux disease. Disease-a-Month. 2020 Jan;66(1):100849. 10.1016/j.disamonth.2019.02.002 30798984

[45] Mintah SO, Asafo-Agyei T, Archer MA, Junior PA, Boamah D, Kumadoh D, et al. Medicinal plants for treatment of prevalent diseases. Pharmacogn-Med Plants. 2019. p. 1–9. 10.5772/intechopen.82049.

[46] Tran N, Pham B, Le L. Bioactive compounds in anti-diabetic plants: From herbal medicine to modern drug discovery. Biology. 2020 Aug;9(9):252. 10.3390/biology9090252 PMC7563488 32872226

[47] Cordero CS, Meve U, Alejandro GJ. Ethnobotanical documentation of medicinal plants used by the indigenous panay bukidnon in lambunao, iloilo, Philippines. Front Pharmacol. 2022 Jan;12:790567. 10.3389/fphar.2021.790567 PMC8784692 35082673

[48] Kang Z, Zhonga Y, Wu T, Huang J, Zhao H, Liu D. Ginsenoside from ginseng: a promising treatment for inflammatory bowel disease. Pharmacol Rep. 2021 Jun;73:700–11. 10.1007/s43440-020-00213-z PMC8180475 33462754

[49] Shaito A, Thuan DT, Phu HT, Nguyen TH, Hasan H, Halabi S, et al. Herbal medicine for cardiovascular diseases: efficacy, mechanisms, and safety. Front Pharmacol. 2020 Apr;11:422. 10.3389/fphar.2020.00422 PMC7155419 32317975

[50] Batiha GE, Teibo JO, Wasef L, Shaheen HM, Akomolafe AP, Teibo TK, et al. A review of the bioactive components and pharmacological properties of Lavandula species. Naunyn-schmiedeberg’s Arch Pharmacol. 2023 May;396(5):877–900. 10.1007/s00210-023-02392-x PMC10079719 36773055

[51] Mangisa M, Kemboi D, Fouche G, Nthambeleni R, Langat MK, Tarirai C, et al. Ethnomedicinal Uses, phytochemistry and pharmacological properties of Suregada genus: a review. Pharmaceuticals. 2023 Sep;16(10):1390. 10.3390/ph16101390 PMC10610488 37895862

[52] Vivanco PG, Taboada P, Coelho A. The Southern European atlantic diet and its supplements: The chemical bases of its anticancer properties. Nutrients. 2023 Oct;15(19):4274. 10.3390/nu15194274 PMC10574233 37836558

[53] Pavić V, Jakovljević M, Molnar M, Jokić S. Extraction of carnosic acid and carnosol from sage (Salvia officinalis L.) leaves by supercritical fluid extraction and their antioxidant and antibacterial activity. Plants. 2019 Jan;8(1):16. 10.3390/plants8010016 PMC6359053 30634542

[54] Gutiérrez-Grijalva EP, Picos-Salas MA, Leyva-López N, Criollo-Mendoza MS, Vazquez-Olivo G, Heredia JB. Flavonoids and phenolic acids from oregano: Occurrence, biological activity and health benefits. Plants. 2017 Dec;7(1):2. 10.3390/plants7010002 PMC5874591 29278371

[55] Al-Snafi AE. Chemical constituents and pharmacological effects of Cynodon dactylon-A review. IOSR J Pharm. 2016;6(7):17–31.

[56] Monari S, Ferri M, Salinitro M, Tassoni A. New insights on primary and secondary metabolite contents of seven italian wild food plants with medicinal applications: A comparative study. Plants. 2023 Sep;12(18):3180. 10.3390/plants12183180 PMC10537336 37765345

[57] Dereli FT, Ilhan M, Belwal T, editors. Novel drug targets with traditional herbal medicines: Scientific and clinical evidence. Cham: Springer International Publishing; 2022. p. 495–512.

[58] Hęś M, Dziedzic K, Górecka D, Jędrusek-Golińska A, Gujska E. Aloe vera (L.) Webb.: natural sources of antioxidants–a review. Plant Foods for Human Nutrition. Dordrecht, Netherlands. Vol. 74; 2019 Sep. p. 255–65 10.1007/s11130-019-00747-5 PMC6684795 31209704

[59] Fuloria S, Mehta J, Chandel A, Sekar M, Rani NN, Begum MY, et al. A comprehensive review on the therapeutic potential of Curcuma longa Linn. in relation to its major active constituent curcumin. Front Pharmacol. 2022 Mar;13:820806. 10.3389/fphar.2022.820806 PMC8990857 35401176

[60] Semwal RB, Semwal DK, Combrinck S, Viljoen AM. Gingerols and shogaols: Important nutraceutical principles from ginger. Phytochemistry. 2015 Sep;117:554–68. 10.1016/j.phytochem.2015.07.012 26228533

[61] Chaachouay N, Belhaj S, El Khomsi M, Benkhnigue O, Zidane L. Herbal remedies used to treat digestive system ailments by indigenous communities in the Rif region of northern Morocco. Vegetos. 2024 Feb;37(1):379–96.

[62] Aziz MA, Adnan M, Khan AH, Shahat AA, Al-Said MS, Ullah R. Traditional uses of medicinal plants practiced by the indigenous communities at Mohmand Agency, FATA, Pakistan. J Ethnobiol Ethnomed. 2018 Dec;14:1–6. 10.1186/s13002-017-0204-5 PMC5761105 29316948

[63] Martinez-Aledo N, Navas-Carrillo D, Orenes-Pinero E. Medicinal plants: active compounds, properties and antiproliferative effects in colorectal cancer. Phytochem Rev. 2020 Feb;19:123–37.

[64] Del Valle J. Peptic ulcer disease and related disorders. Harrisons Princ Intern Med. 2005;16(2):1746.

[65] Lanas A, Chan FK. Peptic ulcer disease. Lancet. 2017 Aug;390(10094):613–24. 10.1016/S0140-6736(16)32404-7 28242110

[66] Rosenstock SJ, Jørgensen T. Prevalence and incidence of peptic ulcer disease in a Danish County--a prospective cohort study. Gut. 1995 Jun;36(6):819–24. 10.1136/gut.36.6.819 PMC1382615 7615266

[67] Kurata JH, Nogawa AN, Abbey DE, Petersen F. A prospective study of risk for peptic ulcer disease in Seventh-Day Adventists. Gastroenterology. 1992 Mar;102(3):902–9. 10.1016/0016-5085(92)90176-y 1537526

[68] McQuaid KR. Drugs used in the treatment of gastrointestinal diseases (Ch. 62). In: Katzung BG, Trevor AJ, editors. Basic and Clinical Pharmacology. 15th edn. San Francisco: McGraw-Hill/Medical; 2020.

[69] Alatab S, Sepanlou SG, Ikuta K, Vahedi H, Bisignano C, Safiri S, et al. The global, regional, and national burden of inflammatory bowel disease in 195 countries and territories, 1990–2017: a systematic analysis for the Global Burden of Disease Study 2017. Lancet Gastroenterol Hepatol. 2020 Jan;5(1):17–30. 10.1016/S2468-1253(19)30333-4 PMC7026709 31648971

[70] Benchimol EI, Mack DR, Guttmann A, Nguyen GC, To T, Mojaverian N, et al. Inflammatory bowel disease in immigrants to Canada and their children: a population-based cohort study. Off J Am Coll Gastroenterol ACG. 2015 Apr;110(4):553–63. 10.1038/ajg.2015.52 25756238

[71] Biller LH, Schrag D. Diagnosis and treatment of metastatic colorectal cancer: a review. Jama. 2021 Feb;325(7):669–85. 10.1001/jama.2021.0106 33591350

[72] Kanth P, Inadomi JM. Screening and prevention of colorectal cancer. Bmj. 2021 Sep;374:n1855. 10.1136/bmj.n1855 34526356

[73] Singh P, Tuck C, Gibson PR, Chey WD. The role of food in the treatment of bowel disorders: focus on irritable bowel syndrome and functional constipation. Am J Gastroenterology. 2022 Jun;117(6):947. 10.14309/ajg.0000000000001767 PMC9169760 35435179

[74] Lovell RM, Ford AC. Global prevalence of and risk factors for irritable bowel syndrome: a meta-analysis. Clin Gastroenterol Hepatol. 2012 Jul;10(7):712–21. 10.1016/j.cgh.2012.02.029 22426087

[75] Lovell RM, Ford AC. Effect of gender on prevalence of irritable bowel syndrome in the community: systematic review and meta-analysis. Off J Am Coll Gastroenterol ACG. 2012 Jul;107(7):991–1000. 10.1038/ajg.2012.131 22613905

[76] Canavan C, West J, Card T. The economic impact of the irritable bowel syndrome. Aliment Pharmacol Ther. 2014 Nov;40(9):1023–34. 10.1111/apt.12938 25199904

[77] Bond A, Huijbers A, Pironi L, Schneider SM, Wanten G, Lal S. Diagnosis and management of intestinal failure‐associated liver disease in adults. Aliment Pharmacol Ther. 2019 Sep;50(6):640–53. 10.1111/apt.15432 31342540

[78] Cui Y, Wang Q, Chang R, Zhou X, Xu C. Intestinal barrier function–non-alcoholic fatty liver disease interactions and possible role of gut microbiota. J Agric Food Chem. 2019 Feb;67(10):2754–62. 10.1021/acs.jafc.9b00080 30798598

[79] Trefts E, Gannon M, Wasserman DH. The liver. Curr Biol. 2017;27(21):R1147–51. 10.1016/j.cub.2017.09.019 PMC5897118 29112863

[80] Asrani SK, Devarbhavi H, Eaton J, Kamath PS. Burden of liver diseases in the world. J Hepatology. 2019 Jan;70(1):151–71. 10.1016/j.jhep.2018.09.014 30266282

[81] Ding L, Li J, Song B, Xiao X, Huang W, Zhang B, et al. Andrographolide prevents high-fat diet–induced obesity in C57BL/6 mice by suppressing the sterol regulatory element-binding protein pathway. J Pharmacol Exp Therapeutics. 2014 Nov;351(2):474–83. 10.1124/jpet.114.217968 25204338

[82] Marjani M, Baghaei P, Dizaji MK, Bayani PG, Fahimi F, Tabarsi P, et al. Evaluation of hepatoprotective effect of silymarin among under treatment tuberculosis patients: a randomized clinical trial. Iran J Pharm Res: IJPR. 2016;15(1):247. PMC4986122 27610165

[83] Saller R, Meier R, Brignoli R. The use of silymarin in the treatment of liver diseases. Drugs. 2001 Dec;61:2035–63. 10.2165/00003495-200161140-00003 11735632

[84] Pandey B, Baral R, Kaundinnyayana A, Panta S. Promising hepatoprotective agents from the natural sources: a study of scientific evidence. Egypt Liver J. 2023 Mar;13(1):14.

[85] Arman M, Chowdhury KA, Bari MS, Khan MF, Huq MM, Haque MA, et al. Hepatoprotective potential of selected medicinally important herbs: evidence from ethnomedicinal, toxicological and pharmacological evaluations. Phytochem Rev. 2022 Dec;21(6):1863–86.

[86] Ugwu CE, Suru SM. Medicinal plants with hepatoprotective potentials against carbon tetrachloride-induced toxicity: a review. Egypt Liver J. 2021 Dec;11(1):1–26.

[87] Son CG, Wei Z, Raghavendran HB, Wang JH, Janda E. Medicinal herbs and their active compounds for fatty liver diseases. Evidence-Based Complementary Altern Med. 2017 Jan;2017:3612478. 10.1155/2017/3612478 PMC5738570 29362587

[88] Fulda S. Betulinic acid for cancer treatment and prevention. Int J Mol Sci. 2008 Jun;9(6):1096–107. 10.3390/ijms9061096 PMC2658785 19325847

[89] Gong JY, Ren H, Peng SY, Xing K, Fan L, Liu MZ, et al. Comparative effectiveness of glycyrrhizic acid preparations aimed at preventing and treating anti-tuberculosis drug-induced liver injury: a network meta-analysis of 97 randomized controlled trials. Phytomedicine. 2022 Apr;98:153942. 10.1016/j.phymed.2022.153942 35093672

[90] Kara E, Coşkun T, Kaya Y, Yumuş O, Vatansever S, Var A. Effects of silymarin and pentoxifylline on matrix metalloproteinase-1 and-2 expression and apoptosis in experimental hepatic fibrosis. Curr Ther Res. 2008 Dec;69(6):488–502. 10.1016/j.curtheres.2008.12.003 PMC3969983 24692823

[91] Sen A. Prophylactic and therapeutic roles of oleanolic acid and its derivatives in several diseases. World J Clin Cases. 2020 May;8(10):1767. 10.12998/wjcc.v8.i10.1767 PMC7262697 32518769

[92] Kataria R, Pickett-Blakely O. The Mediterranean Diet in Gastrointestinal and liver diseases. Curr Treat Options Gastroenterol. 2020 Dec;18:718–28.

[93] Yao Y, Habib M, Bajwa HF, Qureshi A, Fareed R, Altaf R, et al. Herbal therapies in gastrointestinal and hepatic disorders: An evidence-based clinical review. Front Pharmacol. 2022 Oct;13:3980. 10.3389/fphar.2022.962095 PMC9581220 36278240

[94] Salmi HA, Sarna S. Effect of silymarin on chemical, functional, and morphological alterations of the liver: a double-blind controlled study. Scand J Gastroenterol. 1982 Jun;17(4):517–21. 10.3109/00365528209182242 6753109

[95] Anushiravani A, Haddadi N, Pourfarmanbar M, Mohammadkarimi V. Treatment options for nonalcoholic fatty liver disease: a double-blinded randomized placebo-controlled trial. Eur J Gastroenterol Hepatol. 2019 May;31(5):613–7. 10.1097/MEG.0000000000001369 30920975

[96] Clichici S, Olteanu D, Nagy AL, Oros A, Filip A, Mircea PA. Silymarin inhibits the progression of fibrosis in the early stages of liver injury in CCl4-treated rats. J Med Food. 2015 Mar;18(3):290–8. 10.1089/jmf.2013.0179 25133972

[97] Zhang P, Ma D, Wang Y, Zhang M, Qiang X, Liao M, et al. Berberine protects liver from ethanol-induced oxidative stress and steatosis in mice. Food Chem Toxicol. 2014 Dec;74:225–32. 10.1016/j.fct.2014.10.005 25455889

[98] Simental-Mendía LE, Pirro M, Gotto AM Jr, Banach M, Atkin SL, Majeed M, et al. Lipid-modifying activity of curcuminoids: A systematic review and meta-analysis of randomized controlled trials. Crit Rev Food Sci Nutr. 2019 Apr;59(7):1178–87. 10.1080/10408398.2017.1396201 29185808

[99] Zendedel E, Butler AE, Atkin SL, Sahebkar A. Impact of curcumin on sirtuins: A review. J Cell Biochem. 2018 Dec;119(12):10291–300. 10.1002/jcb.27371 30145851

[100] Lee ES, Kwon MH, Kim HM, Woo HB, Ahn CM, Chung CH. Curcumin analog CUR5–8 ameliorates nonalcoholic fatty liver disease in mice with high-fat diet-induced obesity. Metabolism. 2020 Feb;103:154015. 10.1016/j.metabol.2019.154015 31758951

[101] Rahmani S, Asgary S, Askari G, Keshvari M, Hatamipour M, Feizi A, et al. Treatment of non‐alcoholic fatty liver disease with curcumin: A randomized placebo‐controlled trial. Phytother Res. 2016 Sep;30(9):1540–8. 10.1002/ptr.5659 27270872

[102] Mirhafez SR, Azimi-Nezhad M, Dehabeh M, Hariri M, Naderan RD, Movahedi A, et al. The effect of curcumin phytosome on the treatment of patients with non-alcoholic fatty liver disease: a double-blind, randomized, placebo-controlled trial. In: Pharmacological properties of plant-derived natural products and implications for human health. Vol. 1308; 2021. p. 25–35. 10.1007/978-3-030-64872-5_3 33861434

[103] Chen G, Bei B, Feng Y, Li X, Jiang Z, Si JY, et al. Glycyrrhetinic acid maintains intestinal homeostasis via HuR. Front Pharmacology. 2019 May;10:535. 10.3389/fphar.2019.00535 PMC6531911 31156441

[104] Kim M, Yang SG, Kim JM, Lee JW, Kim YS, Lee JI. Silymarin suppresses hepatic stellate cell activation in a dietary rat model of non-alcoholic steatohepatitis: Analysis of isolated hepatic stellate cells. Int J Mol Med. 2012 Sep;30(3):473–9. 10.3892/ijmm.2012.1029 PMC3573753 22710359

[105] Harish R, Shivanandappa T. Antioxidant activity and hepatoprotective potential of Phyllanthus niruri. Food Chem. 2006 Mar;95(2):180–5.

[106] Lin CM, Lee JF, Chiang LL, Chen CF, Wang D, Su CL. The protective effect of curcumin on ischemia-reperfusion–induced liver injury. In Transplantation proceedings. Vol. 44. Issue 4. USA: Elsevier; 2012 May. p. 974–7. 10.1016/j.transproceed.2012.01.081 22564600

[107] Antunes C, Arbo MD, Konrath EL. Hepatoprotective native plants documented in Brazilian traditional medicine literature: Current knowledge and prospects. Chem & Biodivers. 2022 Jun;19(6):e202100933. 10.1002/cbdv.202100933 35421282

[108] Ahmad A, Ahmad R. Resveratrol mitigate structural changes and hepatic stellate cell activation in N′-nitrosodimethylamine-induced liver fibrosis via restraining oxidative damage. Chem-Biol Interact. 2014 Sep;221:1–2. 10.1016/j.cbi.2014.07.007 25064540

[109] Koutelidakis A, Dimou C. The effects of functional food and bioactive compounds on biomarkers of cardiovascular diseases. In: Martirosyan D, editor. Functional foods in health and disease; USA. 1st edn. 2016. p. 89–117.

[110] Kwak NS, Jukes DJ. Functional foods. Part 2: the impact on current regulatory terminology. Food Control. 2001 Mar;12(2):109–17.

[111] Trichopoulou A, Bamia C, Trichopoulos D. Anatomy of health effects of Mediterranean diet: Greek EPIC prospective cohort study. Bmj. 2009 Jun;338:b2337. 10.1136/bmj.b2337 PMC3272659 19549997

[112] García-Montero C, Fraile-Martinez O, Gomez-Lahoz AM, Pekarek L, Castellanos AJ, et al. Nutritional components in Western diet versus Mediterranean diet at the gut microbiota–immune system interplay. Implications for health and disease. Nutrients. 2021 Feb;13(2):699. 10.3390/nu13020699 PMC7927055 33671569

[113] Vanuytsel T, Van Wanrooy S, Vanheel H, Vanormelingen C, Verschueren S, Houben E, et al. Psychological stress and corticotropin-releasing hormone increase intestinal permeability in humans by a mast cell-dependent mechanism. Gut. 2014 Aug;63(8):1293–9. 10.1136/gutjnl-2013-305690 24153250

[114] De la Roca-Chiapas JM, Solís-Ortiz S, Fajardo-Araujo M, Sosa M, Córdova-Fraga T, Rosa-Zarate A. Stress profile, coping style, anxiety, depression, and gastric emptying as predictors of functional dyspepsia: a case-control study. J Psychosom Res. 2010 Jan;68(1):73–81. 10.1016/j.jpsychores.2009.05.013 20004303

[115] Gentschew L, Ferguson LR. Role of nutrition and microbiota in susceptibility to inflammatory bowel diseases. Mol Nutr Food Res. 2012 Apr;56(4):524–35. 10.1002/mnfr.201100630 22495981

[116] Valdes AM, Walter J, Segal E, Spector TD. Role of the gut microbiota in nutrition and health. Bmj. 2018 Jun;361. 10.1136/bmj.k2179 PMC6000740 29899036

[117] Santonicola A, Gagliardi M, Guarino MP, Siniscalchi M, Ciacci C, Iovino P. Eating disorders and gastrointestinal diseases. Nutrients. 2019 Dec;11(12):3038. 10.3390/nu11123038 PMC6950592 31842421

[118] De Luca I, Di Cristo F, Valentino A, Peluso G, Di Salle A, Calarco A. Food-derived bioactive molecules from mediterranean diet: nanotechnological approaches and waste valorization as strategies to improve human wellness. Polymers. 2022 Apr;14(9):1726. 10.3390/polym14091726 PMC9103748 35566894

[119] Menotti A, Puddu PE, Catasta G. Dietary habits, cardiovascular and other causes of death in a practically extinct cohort of middle-aged men followed-up for 61 years. Nutr Metab Cardiovasc Dis. 2022 Aug;32(8):1819–29. 10.1016/j.numecd.2022.04.010 35599088

[120] Jenkins D, Modolell I. Proton pump inhibitors. BMJ. 2023 Nov;383:e070752. 10.1136/bmj-2022-070752 37957000

[121] Voropaiev M, Nock D. Onset of acid-neutralizing action of a calcium/magnesium carbonate-based antacid using an artificial stomach model: an in vitro evaluation. BMC Gastroenterol. 2021 Dec;21(1):1–6. 10.1186/s12876-021-01687-8 PMC7937289 33676393

[122] El Rouby N, Lima JJ, Johnson JA. Proton pump inhibitors: from CYP2C19 pharmacogenetics to precision medicine. Expert Opin Drug Metab Toxicol. 2018 Apr;14(4):447–60. 10.1080/17425255.2018.1461835 PMC5942154 29620484

[123] Modak AS, Klyarytska I, Kriviy V, Tsapyak T, Rabotyagova Y. The effect of proton pump inhibitors on the CYP2C19 enzyme activity evaluated by the pantoprazole-13C breath test in GERD patients: clinical relevance for personalized medicine. J Breath Res. 2016 Dec;10(4):046017. 10.1088/1752-7163/10/4/046017 27991432

[124] Arya AK, Durgapal M, Bachheti A, Joshi KK, Gonfa YH, Bachheti RK, et al. Ethnomedicinal use, phytochemistry, and other potential application of aquatic and semiaquatic medicinal plants. Evidence-Based Complementary Altern Med. 2022 Aug;2022:4931556. 10.1155/2022/4931556 PMC9385301 35990854

[125] Saleh HA, Yousef MH, Abdelnaser A. The anti-inflammatory properties of phytochemicals and their effects on epigenetic mechanisms involved in TLR4/NF-κB-mediated inflammation. Front Immunol. 2021 Mar;12:606069. 10.3389/fimmu.2021.606069 PMC8044831 33868227

[126] Yu C, Wang D, Yang Z, Wang T. Pharmacological effects of polyphenol phytochemicals on the intestinal inflammation via targeting TLR4/NF-κB signaling pathway. Int J Mol Sci. 2022 Jun;23(13):6939. 10.3390/ijms23136939 PMC9266441 35805952

[127] Lillehoj H, Liu Y, Calsamiglia S, Fernandez-Miyakawa ME, Chi F, Cravens RL, et al. Phytochemicals as antibiotic alternatives to promote growth and enhance host health. Vet Res. 2018 Dec;49(1):1–8. 10.1186/s13567-018-0562-6 PMC6066919 30060764

[128] El Amrousy D, Elashry H, Salamah A, Maher S, Abd-Elsalam SM, Hasan S. Adherence to the mediterranean diet improved clinical scores and inflammatory markers in children with active inflammatory bowel disease: A randomized trial. J Inflamm Res. 2022 Mar;2022:2075–86. 10.2147/JIR.S349502 PMC8994055 35411169

[129] Fürst R, Zündorf I. Plant-derived anti-inflammatory compounds: hopes and disappointments regarding the translation of preclinical knowledge into clinical progress. Mediators Inflamm. 2014 Oct;2014:146832. 10.1155/2014/146832 PMC4060065 24987194

[130] Cho SY, Park SJ, Kwon MJ, Jeong TS, Bok SH, Choi WY, et al. Quercetin suppresses proinflammatory cytokines production through MAP kinases and NF-κB pathway in lipopolysaccharide-stimulated macrophage. Mol Cell Biochem. 2003 Jan;243:153–60. 10.1023/a:1021624520740 12619901

[131] Shao D, Lian Z, Di Y, Zhang L, Rajoka MS, Zhang Y, et al. Dietary compounds have potential in controlling atherosclerosis by modulating macrophage cholesterol metabolism and inflammation via miRNA. npj Science of Food. 2018 Jul;2(1):13. 10.1038/s41538-018-0022-8 PMC6550192 31304263

[132] Aggarwal BB, Kumar A, Bharti AC. Anticancer potential of curcumin: preclinical and clinical studies. Anticancer Res. 2003 Jan;23(1/A):363–98. 12680238

[133] Menon VP, Sudheer AR. Antioxidant and anti-inflammatory properties of curcumin. In: The molecular targets and therapeutic uses of Curcumin in health and disease. Vol. 595; 2007 Jan; p. 105–25. 10.1007/978-0-387-46401-5_3 17569207

[134] Lubbad A, Oriowo MA, Khan I. Curcumin attenuates inflammation through inhibition of TLR-4 receptor in experimental colitis. Mol Cell Biochem. 2009 Feb;322:127–35. 10.1007/s11010-008-9949-4 19002562

[135] Guimarães MR, Leite FR, Spolidorio LC, Kirkwood KL, Rossa C Jr. Curcumin abrogates LPS-induced pro-inflammatory cytokines in RAW 264.7 macrophages. Evidence for novel mechanisms involving SOCS-1,-3 and p38 MAPK. Arch Oral Biol. 2013 Oct;58(10):1309–17. 10.1016/j.archoralbio.2013.07.005 PMC4030384 24011306

[136] Sandner G, König A, Wallner M, Weghuber J. Functional foods-dietary or herbal products on obesity: Application of selected bioactive compounds to target lipid metabolism. Curr Opin Food Sci. 2020 Aug;34:9–20.

[137] Hatcher H, Planalp R, Cho J, Torti FM, Torti SV. Curcumin: from ancient medicine to current clinical trials. Cell Mol Life Sci. 2008 Jun;65:1631–52. 10.1007/s00018-008-7452-4 PMC4686230 18324353

[138] Tu CT, Han B, Yao QY, Zhang YA, Liu HC, Zhang SC. Curcumin attenuates Concanavalin A-induced liver injury in mice by inhibition of Toll-like receptor (TLR) 2, TLR4 and TLR9 expression. Int Immunopharmacol. 2012 Jan;12(1):151–7. 10.1016/j.intimp.2011.11.005 22138522

[139] Ma F, Liu F, Ding L, You M, Yue H, Zhou Y, et al. Anti-inflammatory effects of curcumin are associated with down regulating microRNA-155 in LPS-treated macrophages and mice. Pharm Biol. 2017 Jan;55(1):1263–73. 10.1080/13880209.2017.1297838 PMC6130682 28264607

[140] Reuter S, Gupta SC, Park B, Goel A, Aggarwal BB. Epigenetic changes induced by curcumin and other natural compounds. Genes Nutr. 2011 May;6:93–108. 10.1007/s12263-011-0222-1 PMC3092901 21516481

[141] Esatbeyoglu T, Huebbe P, Ernst IM, Chin D, Wagner AE, Rimbach G. Curcumin—from molecule to biological function. Angew Chem Int Ed. 2012 May;51(22):5308–32. 10.1002/anie.201107724 22566109

[142] Lichtenstein GR, Hanauer SB, Sandborn WJ. Emerging treatment options in mild to moderate ulcerative colitis. Gastroenterol Hepatol. 2015 Mar;11(3 Suppl 1):1. PMC4609145 26491415

[143] Rahmani AH, Alsahli MA, Aly SM, Khan MA, Aldebasi YH. Role of curcumin in disease prevention and treatment. Adv Biomed Res. 2018;7:38. 10.4103/abr.abr_147_16 PMC5852989 29629341

[144] Ferraz CR, Carvalho TT, Manchope MF, Artero NA, Rasquel-Oliveira FS, Fattori V, et al. Therapeutic potential of flavonoids in pain and inflammation: mechanisms of action, pre-clinical and clinical data, and pharmaceutical development. Molecules. 2020 Feb;25(3):762. 10.3390/molecules25030762 PMC7037709 32050623

[145] Trinchet JC, Coste T, Levy VG, Vivet F, Duchatelle V, Legendre C. Traitement de l’hépatite alcoolique par la silymarine. Une étude comparative en double insu chez 116 malades. Gastroentérologie Clin et Biologique. 1989;13(2):120–4. 2707520

[146] Saad B, Ghareeb B, Kmail A. Metabolic and epigenetics action mechanisms of antiobesity medicinal plants and phytochemicals. Evidence-based Complementary Altern Med. 2021 Jun;2021:1–9. 10.1155/2021/9995903 PMC8208872 34211580

[147] Mannino G, Gentile C, Ertani A, Serio G, Bertea CM. Anthocyanins: Biosynthesis, distribution, ecological role, and use of biostimulants to increase their content in plant foods – A review. Agriculture. 2021 Mar;11(3):212.

[148] Cui HX, Chen JH, Li JW, Cheng FR, Yuan K. Protection of anthocyanin from Myrica rubra against cerebral ischemia-reperfusion injury via modulation of the TLR4/NF-κB and NLRP3 pathways. Molecules. 2018 Jul;23(7):1788. 10.3390/molecules23071788 PMC6099489 30036952

[149] Aboonabi A, Singh I. Chemopreventive role of anthocyanins in atherosclerosis via activation of Nrf2–ARE as an indicator and modulator of redox. Biomed Pharmacother. 2015 May;72:30–6. 10.1016/j.biopha.2015.03.008 26054672

[150] Wei J, Zhang G, Zhang X, Xu D, Gao J, Fan J. Anthocyanins delay ageing-related degenerative changes in the liver. Plant Foods Hum Nutr. 2017 Dec;72:425–31. 10.1007/s11130-017-0644-z 29075987

[151] Cappellini F, Marinelli A, Toccaceli M, Tonelli C, Petroni K. Anthocyanins: from mechanisms of regulation in plants to health benefits in foods. Front Plant Sci. 2021 Oct;12:748049. 10.3389/fpls.2021.748049 PMC8580863 34777426

[152] Lauwers GY, Langner C. Inflammatory and infectious pathology of the gastrointestinal tract: an introduction. Virchows Arch. 2018 Jan;472:1–2. 10.1007/s00428-017-2284-y 29282536

[153] Khare T, Anand U, Dey A, Assaraf YG, Chen ZS, Liu Z, et al. Exploring phytochemicals for combating antibiotic resistance in microbial pathogens. Front Pharmacology. 2021 Jul;12:720726. 10.3389/fphar.2021.720726 PMC8334005 34366872

[154] Tiku AR. Antimicrobial compounds (phytoanticipins and phytoalexins) and their role in plant defense. Co-Evolution Secondary Metabolites; 2020. p. 845–68.

[155] Biharee A, Sharma A, Kumar A, Jaitak V. Antimicrobial flavonoids as a potential substitute for overcoming antimicrobial resistance. Fitoterapia. 2020 Oct;146:104720. 10.1016/j.fitote.2020.104720 32910994

[156] Rahman SA, Abd-Ellatif SA, Deraz SF, Khalil AA. Antibacterial activity of some wild medicinal plants collected from western Mediterranean coast, Egypt: Natural alternatives for infectious disease treatment. Afr J Biotechnol. 2011;10(52):10733–43.

[157] Sivapriya M, Dinesha R, Harsha R, Gowda SS, Srinivas L. Antibacterial activity of different extracts of Sundakai (Solanum torvum) fruit coat. Int J Biol Chem. 2011;5(1):61–7.

[158] Díaz-Nuñez JL, García-Contreras R, Castillo-Juárez I. The new antibacterial properties of the plants: Quo vadis studies of anti-virulence phytochemicals? Front Microbiol. 2021 May;12:667126. 10.3389/fmicb.2021.667126 PMC8137972 34025622

[159] Mattioli R, Francioso A, Mosca L, Silva P. Anthocyanins: A comprehensive review of their chemical properties and health effects on cardiovascular and neurodegenerative diseases. Molecules. 2020 Aug;25(17):3809. 10.3390/molecules25173809 PMC7504512 32825684

[160] Williams M, McCarthy D, Foster A, Yednock SF, Rydel R, Messersmith E, et al. Comprehensive medicinal chemistry II Volume 6: Therapeutic Areas I: Central nervous system, pain, metabolic syndrome, urology, gastrointestinal and cardiovascular. Amsterdam, Netherlands: Elsevier; 2007.

[161] Rauf A, Akram M, Semwal P, Mujawah AA, Muhammad N, Riaz Z, et al. Antispasmodic potential of medicinal plants: a comprehensive review. Oxid Med Cell Longev. 2021 Nov;2021:4889719. 10.1155/2021/4889719 PMC8601825 34804367

[162] Dereli FT. Plant-based bioactive components: Phytochemicals: A review. Bioactive components: A sustainable system for good health and well-being. Singapore: Springer; 2022 Dec. p. 27–33.

[163] Annaházi A, Róka R, Rosztóczy A, Wittmann T. Role of antispasmodics in the treatment of irritable bowel syndrome. World J Gastroenterol: WJG. 2014 May;20(20):6031. 10.3748/wjg.v20.i20.6031 PMC4033443 24876726

[164] Baiu I, Hawn MT. Gallstones and biliary colic. JAMA. 2018 Oct;320(15):1612. 10.1001/jama.2018.11868 30326127

[165] Sanagapalli S, Agnihotri K, Leong R, Corte CJ. Antispasmodic drugs in colonoscopy: a review of their pharmacology, safety and efficacy in improving polyp detection and related outcomes. Ther Adv Gastroenterol. 2017 Jan;10(1):101–13. 10.1177/1756283X16670076 PMC5330606 28286563

[166] Shi X, Chen Z, Yang Y, Yan S. Bile reflux gastritis: insights into pathogenesis, relevant factors, carcinomatous risk, diagnosis, and management. Gastroenterol Res Pract. 2022;2022:2642551. 10.1155/2022/2642551 PMC9484982 36134174

[167] Siddiqui AJ, Jahan S, Singh R, Saxena J, Ashraf SA, Khan A, et al. Plants in anticancer drug discovery: from molecular mechanism to chemoprevention. BioMed Res Int. 2022 Mar;2022:5425485. 10.1155/2022/5425485 PMC8906971 35281598

[168] Skolnick P, Paul SM. Benzodiazepines and nonbenzodiazepines. In Receptor binding in drug research. Boca Raton, Florida, USA: CRC Press; 2020 Aug. p. 53–75

[169] Bourin M. Mechanisms of action of anxiolytics. Psychiatry neurosci update: Epistemology clin psychiatry–Vol IV. Vol. 4; 2021. p. 195–211.

[170] Kenda M, Kočevar Glavač N, Nagy M, Sollner Dolenc M. Medicinal plants used for anxiety, depression, or stress treatment: An update. Molecules. 2022 Sep;27(18):6021. 10.3390/molecules27186021 PMC9500625 36144755

[171] Nallusamy S, Mannu J, Ravikumar C, Angamuthu K, Nathan B, Nachimuthu K, et al. Exploring phytochemicals of traditional medicinal plants exhibiting inhibitory activity against main protease, spike glycoprotein, RNA-dependent RNA polymerase and non-structural proteins of SARS-CoV-2 through virtual screening. Front Pharmacol. 2021 Jul;12:667704. 10.3389/fphar.2021.667704 PMC8295902 34305589

[172] Flores-Bazán T, Betanzos-Cabrera G, Guerrero-Solano JA, Negrete-Díaz JV, German-Ponciano LJ, Olivo-Ramírez D. Pomegranate (Punica granatum L.) and its phytochemicals as anxiolytic; an underreported effect with therapeutic potential: a systematic review. Brain Res. 2023 Aug;1820:148554. 10.1016/j.brainres.2023.148554 37640097

[173] Ali MY, Sina AA, Khandker SS, Neesa L, Tanvir EM, Kabir A, et al. Nutritional composition and bioactive compounds in tomatoes and their impact on human health and disease: A review. Foods. 2020 Dec;10(1):45. 10.3390/foods10010045 PMC7823427 33375293

[174] Gutiérrez-del-Río I, López-Ibáñez S, Magadán-Corpas P, Fernández-Calleja L, Pérez-Valero Á, Tuñón-Granda M, et al. Terpenoids and polyphenols as natural antioxidant agents in food preservation. Antioxidants. 2021 Aug 8;10(8):1264. 10.3390/antiox10081264 PMC8389302 34439512

[175] Kmail A. Protective Role of Hypericum perforatum L. and Hypericum triquetrifolium Turra against Inflammatory Diseases: Evidence from in vitro and in vivo studies. Eur J Med Plants. 2022 Dec;33(12):34–47.

[176] Sen S, Chakraborty R. Food in health preservation and promotion: A special focus on the interplay between oxidative stress and pro-oxidant/antioxidant. Explor Nutr Health Benefits Funct Foods. Boston, USA: IGI Global; 2017. p. 265–300.

[177] Nunes CD, Barreto Arantes M, Menezes de Faria Pereira S, Leandro da Cruz L, de Souza Passos M, Pereira de Moraes, et al. Plants as sources of anti-inflammatory agents. Molecules. 2020 Aug;25(16):3726. 10.3390/molecules25163726 PMC7465135 32824133

[178] Ibrahim R, Barron D. Phenylpropanoids. In Methods in plant biochemistry. Vol. 1. Springer, Boston, MA: Academic Press; 1989 Jan. p. 75–111.

[179] Lin X, Bai D, Wei Z, Zhang Y, Huang Y, Deng H, et al. Curcumin attenuates oxidative stress in RAW264. 7 cells by increasing the activity of antioxidant enzymes and activating the Nrf2-Keap1 pathway. PLoS One. 2019 May;14(5):e0216711. 10.1371/journal.pone.0216711 PMC6528975 31112588

[180] Zebeaman M, Tadesse MG, Bachheti RK, Bachheti A, Gebeyhu R, Chaubey KK. Plants and plant-derived molecules as natural immunomodulators. Bio Med Res Int. 2023 Jun;2023:7711297. 10.1155/2023/7711297 PMC10260316 37313550

[181] Di Sotto A, Vitalone A, Di Giacomo S. Plant-derived nutraceuticals and immune system modulation: an evidence-based overview. Vaccines. 2020 Aug;8(3):468. 10.3390/vaccines8030468 PMC7563161 32842641

[182] Alhazmi HA, Najmi A, Javed SA, Sultana S, Al Bratty M, Makeen HA, et al. Medicinal plants and isolated molecules demonstrating immunomodulation activity as potential alternative therapies for viral diseases including COVID-19. Front Immunol. 2021 May;12:637553. 10.3389/fimmu.2021.637553 PMC8155592 34054806

[183] Rahman MA, Hannan MA, Dash R, Rahman MH, Islam R, Uddin MJ, et al. Phytochemicals as a complement to cancer chemotherapy: Pharmacological modulation of the autophagy-apoptosis pathway. Front Pharmacol. 2021 May;12:639628. 10.3389/fphar.2021.639628 PMC8138161 34025409

[184] Ferdous UT, Yusof ZN. Medicinal prospects of antioxidants from algal sources in cancer therapy. Front Pharmacol. 2021 Mar;12:593116. 10.3389/fphar.2021.593116 PMC7973026 33746748

[185] Sen S, Chakraborty R. Herbs gastrointestinal protection, and oxidative stress. In Gastrointestinal Tissue. Cambridge, Massachusetts: Academic Press; 2017 Jan. p. 259–74.

[186] Domitrović R, Potočnjak I. A comprehensive overview of hepatoprotective natural compounds: mechanism of action and clinical perspectives. Arch Toxicol. 2016 Jan;90:39–79. 10.1007/s00204-015-1580-z 26377694

[187] Garcia-Cortes M, Robles-Diaz M, Stephens C, Ortega-Alonso A, Lucena MI, Andrade RJ. Drug induced liver injury: an update. Arch Toxicol. 2020 Oct;94:3381–407. 10.1007/s00204-020-02885-1 32852569

[188] Martinez-Lacoba R, Pardo-Garcia I, Amo-Saus E, Escribano-Sotos F. Mediterranean diet and health outcomes: A systematic meta-review. Eur J Public Health. 2018 Oct;28(5):955–61. 10.1093/eurpub/cky113 29992229

[189] AlAufi NS, Chan YM, Waly MI, Chin YS, Mohd Yusof BN, Ahmad N. Application of mediterranean diet in cardiovascular diseases and type 2 diabetes mellitus: Motivations and challenges. Nutrients. 2022 Jul;14(13):2777. 10.3390/nu14132777 PMC9268986 35807957

[190] Guasch‐Ferré M, Willett WC. The Mediterranean diet and health: A comprehensive overview. J Intern Med. 2021 Sep;290(3):549–66. 10.1111/joim.13333 34423871

[191] Moustafa B, Trifan G, Isasi CR, Lipton RB, Sotres-Alvarez D, Cai J, et al. Association of Mediterranean diet with cognitive decline among diverse hispanic or latino adults from the Hispanic Community Health Study/Study of Latinos. JAMA Netw Open. 2022 Jul;5(7):e2221982. 10.1001/jamanetworkopen.2022.21982 PMC9284337 35834250

[192] Tosti V, Bertozzi B, Fontana L. Health benefits of the Mediterranean diet: metabolic and molecular mechanisms. J Gerontology: Ser A. 2018 Mar;73(3):318–26. 10.1093/gerona/glx227 PMC7190876 29244059

[193] H. Al Wattar B, Dodds J, Placzek A, Beresford L, Spyreli E, Moore A, et al. Mediterranean-style diet in pregnant women with metabolic risk factors (ESTEEM): A pragmatic multicentre randomised trial. PLoS Med. 2019 Jul;16(7):e1002857. 10.1371/journal.pmed.1002857 PMC6650045 31335871

[194] Mone I, Kraja B, Bregu A, Duraj V, Sadiku E, Hyska J, et al. Adherence to a predominantly Mediterranean diet decreases the risk of gastroesophageal reflux disease: a cross-sectional study in a South Eastern European population. Dis Esophagus. 2016 Oct;29(7):794–800. 10.1111/dote.12384 26175057

[195] Senouci F, Ababou A, Chouieb M. Ethnobotanical survey of the medicinal plants used in the Southern Mediterranean. Case study: the region of Bissa (Northeastern Dahra Mountains, Algeria). Pharmacogn J. 2019;11(4):647–59.

[196] Aron-Wisnewsky J, Warmbrunn MV, Nieuwdorp M, Clément K. Metabolism and metabolic disorders and the microbiome: the intestinal microbiota associated with obesity, lipid metabolism, and metabolic health – pathophysiology and therapeutic strategies. Gastroenterology. 2021 Jan;160(2):573–99. 10.1053/j.gastro.2020.10.057 33253685

